# Pomegranate activates TFEB to promote autophagy-lysosomal fitness and mitophagy

**DOI:** 10.1038/s41598-018-37400-1

**Published:** 2019-01-24

**Authors:** Sijie Tan, Chye Yun Yu, Zhi Wei Sim, Zun Siong Low, Brianna Lee, Faith See, Nyo Min, Archana Gautam, Justin Jang Hann Chu, Kee Woei Ng, Esther Wong

**Affiliations:** 1 School of Biological Sciences, Nanyang Technological University, Singapore, 637551 Singapore; 2 Department of Microbiology, National University of Singapore, Singapore, 117545 Singapore; 3 School of Materials Sciences and Engineering, Nanyang Technological University, Singapore, 639798 Singapore; 4 Department of Physiology and Medical Science Cluster, Yong Loo Lin School of Medicine, National University of Singapore, Singapore, 117593 Singapore; 5 Centre for Healthy Ageing, National University Health System, Singapore, 117456 Singapore

## Abstract

Mitochondrial dysfunction underscores aging and diseases. Mitophagy (*mito*chondria + auto*phagy*) is a quality control pathway that preserves mitochondrial health by targeting damaged mitochondria for autophagic degradation. Hence, molecules or compounds that can augment mitophagy are therapeutic candidates to mitigate mitochondrial-related diseases. However, mitochondrial stress remains the most effective inducer of mitophagy. Thus, identification of mitophagy-inducing regimes that are clinically relevant is favorable. In this study, pomegranate extract (PE) supplementation is shown to stimulate mitophagy. PE activates transcription factor EB (TFEB) to upregulate the expression of autophagy and lysosomal genes for mitochondrial quality control under basal and stress conditions. Basally, PE alters mitochondrial morphology and promotes recruitment of autophagosomes to the mitochondria (mitophagosome formation). Upon onset of mitochondrial stress, PE further augments mitophagosome formation, and engages PINK1 and Parkin to the mitochondria to potentiate mitophagy. This cellular phenomenon of PE-induced mitophagy helps to negate superfluous mitochondrial reactive oxygen species (ROS) production and mitochondrial impairment. Overall, our study highlights the potential of PE supplementation as a physiological therapy to modulate TFEB activity to alleviate mitochondrial dysfunction in aging and mitochondrial-related diseases.

## Introduction

Healthy mitochondria are pivotal for cellular homeostasis and survival^1^. Mitophagy is a fundamental process critical for maintaining mitochondrial health by selectively targeting the damaged mitochondria for lysosomal degradation^2^. Growing evidence suggests that dysregulation of mitophagy underscores the pathogenesis of aging and age-related disorders like neurodegeneration^3,4^. Hence, enhancing mitophagy is a prospective therapy to mitigate aging and diseases. Currently, mitophagy is mainly known to be induced by mitochondrial stress^5^. Avenues that are clinically relevant to augment mitophagy remain limited thus far.

Consumption of polyphenols-enriched functional food (food that benefits health beyond basic nutritional function) has been widely researched as a health promoting measure for longevity and disease therapeutics^6^. Pomegranate is a known functional food with multifaceted health benefits^7–9^. Recent studies demonstrate that pomegranate extract (PE) supplementation extends lifespan in *Caenorhabditis elegans* (*C. elegans*)^10^ and drosophila^11^. Notably, nutritional supplementation with urolithin A, a metabolite of pomegranate-associated polyphenol ellagitannins, has been shown to enhance mitophagy^12^. This highlights the possibility of dietary modulation via pomegranate supplementation as a physiological way to potentiate mitophagy.

Transcription factor EB (TFEB) is a master regulator of autophagy and lysosomal genes^13^. Accumulating evidence has highlighted TFEB as a regulator of mitochondrial homeostasis in part by modulating autophagy and mitophagy transcriptome^14^. Interestingly, recent studies support a role of polyphenols in influencing TFEB activation^15,16^. However, the precise mechanism directly linking polyphenols or functional food to mitophagy remains inadequately understood.

In this study, we investigated the effects of PE supplementation on cellular autophagy and TFEB activity, as well as the ability of PE to mediate mitophagy. Here, we present evidence that PE upregulates autophagy via TFEB activation. Interestingly, PE-induced TFEB activation represents a novel mechanism that is independent of the known extracellular signal–regulated kinases 1 and 2 (ERK1/2)^17^, mammalian target of rapamycin complex 1 (mTORC1)^17–19^ and calcineurin^20^ regulatory pathways. Elucidation of the functional significance of PE-induced TFEB activation and autophagy reveals that PE partakes in mitochondrial quality control. PE alters mitochondrial morphology basally to form “donut-shaped” mitochondria that enwrap cytoplasm. PE-induced spherical mitochondria show enhanced recruitment of autophagosomes without increasing basal mitochondrial turnover. Such mitochondrial structural transformation that favorably engages autophagosomes may make the mitochondria highly competent for rapid mitophagosome formation to potentiate PINK1-Parkin mitophagy upon onset of mitochondrial stress. This cellular phenomenon subsequently protects the mitochondria against redox toxicity and dysfunction. Taken together, our work identifies PE as a TFEB activator to expand the autophagy-lysosomal compartments to basally prime mitochondria in close proximity to autophagosomes for facilitating timely mitophagy when needed; and further promotes PINK1-Parkin mitophagy for preservation of mitochondrial health and fitness during cellular stress. This highlights the potential of PE supplementation as a physiological intervention for mitochondrial-related diseases.

## Results

### PE upregulates autophagy

**Figure 1:**
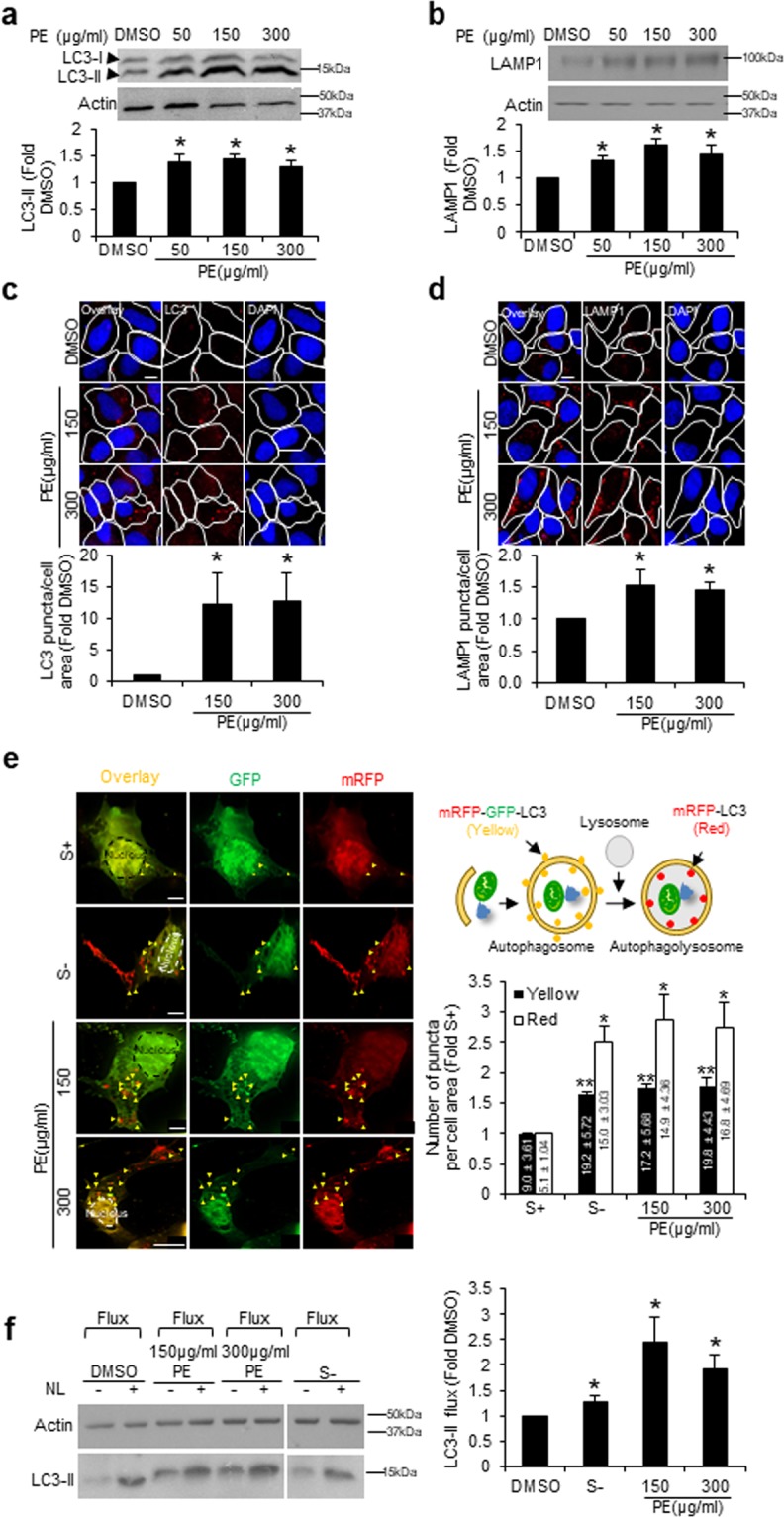
PE upregulates autophagic compartments and autophagy activity in SY5Y cells. (**a,b**) Top: Immunoblots of LC3 (**a**) and LAMP1 (**b**) in SY5Y cells treated with vehicle control DMSO and increasing concentrations of PE at 50, 150 and 300 μg/ml for 24 h. Bottom: Quantification of LC3-II and LAMP1 levels, calculated as fold change against DMSO control. Full-length blots are presented in Supplementary Figure S6a,b. (**c,d**) Top: Immunofluorescence images of endogenous LC3 (**c**) and LAMP1 (**d**) puncta in SY5Y cells treated with DMSO, 150 or 300 μg/ml PE for 24 h. The white outlines highlight the cell shape. Bottom: Quantification of LC3 (**c**) and LAMP1 (**d**) puncta per cell area, calculated as fold change against DMSO control. (**e**) Left: Fluorescence images of yellow (autophagosomes) and red (autophagolysosomes) puncta in SY5Y cells transfected with tandem mRFP-GFP-LC3 reporter for 24 h, followed by 24 h incubation under basal serum containing (S+), serum starvation (S−) or treatment with 150 or 300 μg/ml PE conditions. The white dotted outlines highlight the cell nucleus. The yellow triangles indicate autophagosomes highlighted by both mRFP and GFP signals. Right: Quantification of yellow and red puncta per cell area, calculated as fold change against S+. The values inside the graph bars represent the total number of yellow or red puncta per cell area ± S.E.M under the respective conditions. (**f**) Left: Immunoblot of LC3 in SY5Y cells treated with DMSO, S− or 150 and 300 μg/ml PE for 24 h, followed by 4 h treatment with or without lysosomal inhibitors (NL: 20 mM ammonium chloride and 100 μM leupeptin). Right: Quantification of LC3-II flux, calculated as fold increase in LC3-II levels in the presence of NL over LC3-IIs level in the absence of NL. Full-length blots are presented in Supplementary Figure S6f. At least 30 cells from random fields were analyzed for each condition for all imaging experiments. Nuclei were stained with DAPI. All values are mean + S.E.M (n = 3–4). Differences against DMSO or S+ control are significant at *p<0.05 and **p < 0.01. Scale bar, 10 μm.

Increasing number of studies identified pomegranate and its polyphenolic compounds as autophagy modulators in several cell types^6,12,21–23^. In this study, we assessed if PE could also upregulate autophagy in neuronal SY5Y cells. First, the levels of autophagosomal marker LC3-II and lysosomal marker LAMP1 were measured via immunoblotting. 24 h treatment with 50, 150 and 300 µg/ml of PE significantly induced ~0.5-fold increase in the steady state levels of LC3-II (Fig. 1a) and lysosomal marker LAMP1 (Fig. 1b). These autophagic responses occurred as early as 6 h for LC3-II (Supplementary Fig. S1a) and 12 h for LAMP1 (Supplementary Fig. S1b). Immunofluorescence staining also revealed a significant increase in the number of LC3 (Fig. 1c) and LAMP1 (Fig. 1d) puncta per cell area in PE-treated cells. To further validate the observed upregulation of autophagic compartments with PE, autophagy was analyzed *in-situ* with electron microscopy (EM). Consistent with the earlier findings, EM analysis revealed a high prevalence of autophagic vacuoles (red) and lysosomes (green) in PE-treated cells (Supplementary Fig. S1c). Taken together, these results strongly indicate that PE upregulates the autophagosomal and lysosomal compartments in neuronal SY5Y cells.

Both autophagy induction and impairment can result in an increase in the levels of autophagic compartments. To differentiate between these two possibilities, the autophagic flux was examined. First, the tandem fluorescent mRFP-GFP-LC3 construct was used to monitor the autophagic flux. Due to the different pH stabilities of mRFP and GFP proteins, GFP loses its fluorescence in the presence of lysosomal acidity but not mRFP. Hence, mRFP-GFP signal (yellow) marks the autophagosome, while mRFP signal (red) alone indicates the autophagolysosome^24^ with acidic pH (Fig. 1e). Upon autophagic induction by starvation (S−), there was a significant ~1.6-fold increase in the yellow and ~2.5-fold increase in the red puncta per cell area (Fig. 1e). Mirror effects were also observed in cells treated with 150 and 300 µg/ml PE (Fig. 1e). This demonstrates the ability of PE to upregulate both autophagosome formation and turnover. The finding was also corroborated by the LC3-II flux analysis. Similar to S− response, both concentrations of PE resulted in significantly higher accumulation of LC3-II upon lysosomal inhibition with ammonium chloride and leupeptin (NL) than DMSO control cells (Fig. 1f), highlighting an enhanced rate of autophagic turnover. Together, PE-mediated upregulation of autophagosomal and lysosomal compartments is a positive response to augment autophagy in SY5Y cells.

### PE activates TFEB

**Figure 2:**
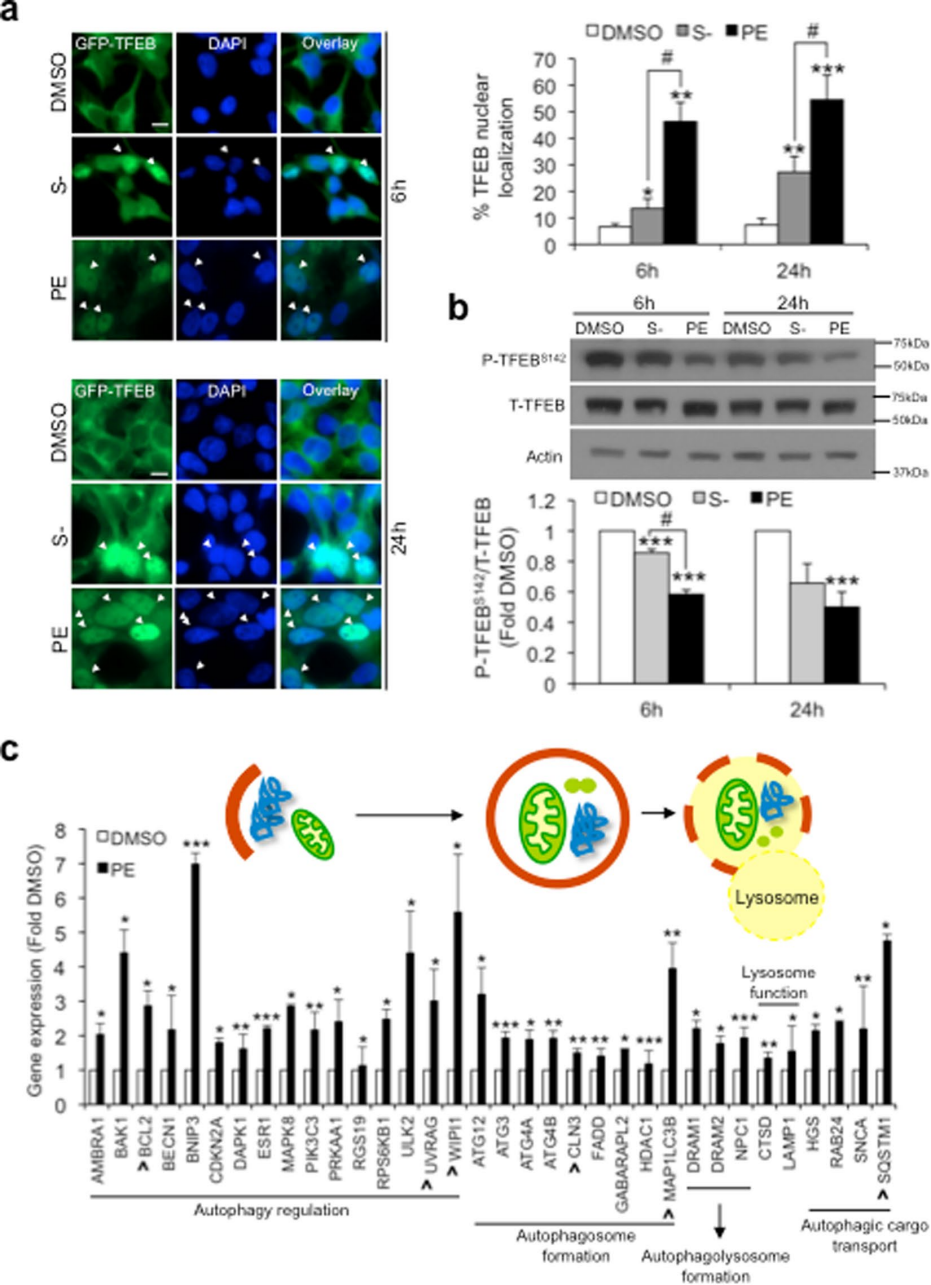
PE promotes TFEB nuclear localization via dephosphorylation at TFEB^S142^ to upregulate autophagy transcriptome. (**a**) Left: Fluorescence images depicting spatial localization of GFP-TFEB in GFP-TFEB SY5Y stable cells treated with DMSO, S− or 300 μg/ml PE for 6 h and 24 h. White arrows indicate cells that contained nuclear-localized GFP-TFEB. Right: Quantification of percentage TFEB nuclear localization. Nuclei were stained with DAPI. At least 100 cells from random fields were analyzed for each condition. (**b**) Top: Immunoblots of phosphorylated TFEB at Ser142 (P-TFEB^S142^) and total TFEB (T-TFEB) in GFP-TFEB SY5Y stable cells treated with DMSO, S− or 300 μg/ml PE for 6 h and 24 h. Bottom: Quantification of P-TFEB^S142^ levels, calculated as fold change against DMSO control. Full-length blots are presented in Supplementary Figure S7b. (**c**) Analysis of real-time PCR autophagy array in SY5Y cells treated with DMSO or 300 μg/ml PE for 24 h. Gene expression was calculated as fold change relative to DMSO control. ^indicates genes that are part of the CLEAR network. All values are mean + S.E.M (n = 3–12). Differences against DMSO or S− are significant at *p<0.05, **p<0.01, ***p<0.005 and ^#^p < 0.05. Scale bar, 10 μm.

TFEB is a master transcription factor that controls autophagy and lysosomal gene expression^13^. Under nutrient-rich condition, TFEB is largely sequestered in the cytosol and kept inactivated^17–19,25^. Upon onset of cellular stress like starvation, TFEB rapidly translocates to the nucleus to activate gene transcription^19,20^. Using the GFP-TFEB SY5Y stable cells, we examined whether PE potentiates autophagy via TFEB activation. 6 h and 24 h S− markedly increased the percentage of cells with nuclear-localized TFEB by more than 1-fold as compared to DMSO control cells (Fig. 2a). PE also significantly enhanced TFEB nuclear shuffling upon 6 h and 24 h treatment (Fig. 2a). Remarkably, both concentrations of PE consistently elicited a stronger TFEB activation response than S− (Fig. 2a).

Spatial localization of TFEB is regulated by phosphorylation. TFEB is sequestered in the cytosol by inhibitory phosphorylation at Ser142 (P-TFEB^S142^)^25^. In line with earlier observations, 6 h and 24 h S− and PE treatment significantly reduced the levels of P-TFEB^S142^ (Fig. 2b). Notably, PE led to a more dramatic reduction in P-TFEB^S142^ levels than S− upon 6 h treatment (Fig. 2b). Taken together, PE activates TFEB nuclear translocation by reducing the inhibitory Ser142 phosphorylation on TFEB. Furthermore, our observations highlight PE as a more potent activator of TFEB than S−.

To affirm that PE-induced TFEB nuclear localization was indeed associated with upregulation of autophagy and lysosomal genes, we performed real-time PCR to profile the gene expression of 84 key human autophagic genes in PE-treated cells (Supplementary Fig. S2). 34 autophagic genes involved in autophagy initiation, autophagosome formation, fusion between autophagosome and lysosome, lysosome function and autophagic cargo transport were significantly upregulated in PE-treated cells (Fig. 2c). Amongst these, *BCL2*, *CLN3*, *MAP1LC3*, *SQTSM1*, *UVRAG*, *WIPI* belong to the Coordinated Lysosomal Expression and Regulation (CLEAR) gene network that is regulated by TFEB (Fig. 2c). This shows that PE-induced TFEB nuclear localization is accompanied by upregulation of TFEB client genes as well as additional autophagy transcriptional programs. Together, our findings strongly highlight PE as an activator of TFEB to upregulate autophagy.

### PE activates TFEB independent of ERK1/2, mTOR and calcineurin

TFEB phosphorylation status is regulated by several kinases and phosphatase. Under nutrient-rich condition, ERK1/2 and mTORC1 mediate inhibitory phosphorylation on TFEB^25^. On the other hand, cellular stressors like starvation induces lysosomal Ca^2+^ release to activate phosphatase calcineurin^20,26^. Calcineurin subsequently dephosphorylates TFEB at Ser142 to induce nuclear shuffling^20^. With the earlier observation that PE activates TFEB, we examined which regulatory mechanism underlies PE-induced TFEB dephosphorylation and activation.

**Figure 3:**
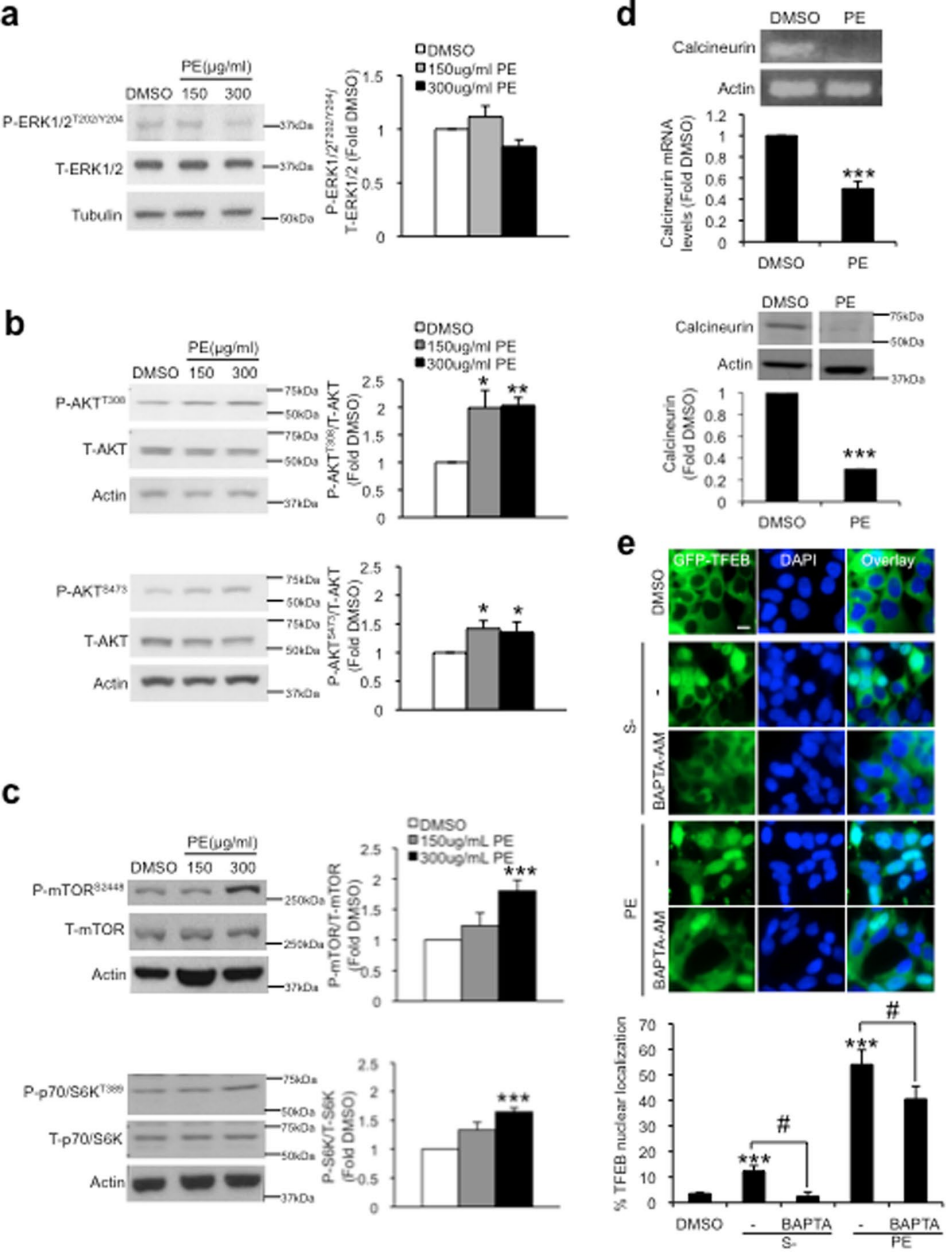
PE does not influence ERK1/2, AKT, mTOR and calcineurin signaling, but regulates TFEB nuclear shuffling in a manner dependent on cytosolic Ca^2+^ levels. (**a–c**) Left: Immunoblots of phosphorylated (P) and total (T) forms of ERK1/2 (**a**), AKT (**b**), mTOR and p70/S6K (**c**) in SY5Y cells treated with DMSO, 150 or 300 μg/ml PE for 16 h. Right: Quantification of the various phosphorylated proteins against their respective total proteins, expressed as fold change relative to DMSO control. Full-length blots are presented in Supplementary Figure S8a–c. (**d**) Top: PCR analysis of calcineurin mRNA expression levels in SY5Y cells after 24 h treatment with DMSO or 300 μg/ml PE. Graph shows quantification of calcineurin mRNA levels, expressed as fold change against DMSO control. Bottom: Immunoblot of calcineurin in SY5Y cells treated with DMSO or 300 μg/ml PE for 16 h. Graph shows quantification of calcineurin levels, calculated as fold change relative to DMSO control. Full-length blots are presented in Supplementary Figure S8d. (**e**) Top: Fluorescence images depicting spatial localization of GFP-TFEB in GFP-TFEB SY5Y stable cells treated with DMSO, starved (S−) in the absence or presence of 10 µM Ca^2+^ chelator BAPTA-AM, or treated with 300 μg/ml PE in the absence or presence of BAPTA-AM for 24 h. Bottom: Quantification of percentage TFEB nuclear localization. Nuclei were stained with DAPI. At least 100 cells from random fields were analyzed for each condition. All values are mean + S.E.M (n = 3–7). Differences against DMSO are significant at *p<0.05, **p<0.01 and ***p<0.005 and ^#^p < 0.05. Scale bar, 10 μm.

150 and 300 µg/ml PE did not influence ERK1/2 phosphorylation and activation (Fig. 3a), highlighting that PE does not affect ERK1/2 signaling and is unlikely to regulate TFEB through this pathway. mTORC1 is phosphorylated and activated by AKT^27^. In turn, AKT is activated by phosphorylation at Thr308 and Ser473 to inhibit autophagy^28^. 150 and 300 µg/ml PE significantly increased the levels of phosphorylated AKT at both sites by ~1-fold (Fig. 3b). Accordingly, an increased in the phosphorylation of mTOR as well as the downstream effector protein ribosomal protein S6 kinase (p70/S6K) were also observed in PE-treated cells (Fig. 3c). These results demonstrate that PE activates AKT-mTORC1 signaling axis, contrary to the earlier observations of enhanced autophagy and TFEB activation with PE.

The Ca^2+^-calcineurin pathway has been reported to activate TFEB independent of mTORC1 activity^20^. This raises the possibly of this pathway in mediating PE-induced TFEB activation. Unexpectedly, the gene expression and protein levels of calcineurin were significantly lowered in PE-treated cells (Fig. 3d), suggesting that calcineurin may be dispensable for PE-induced TFEB activation. Moreover, addition of calcineurin inhibitor FK506 at concentration that significantly reduced S− induced TFEB nuclear localization did not perturb PE-induced TFEB nuclear shuffling (Supplementary Fig. S3). Thus, activation of TFEB by PE is independent of calcineurin as PE inhibits gene transcription of calcineurin.

Although PE-induced TFEB activation is independent of calcineurin, addition of Ca^2+^ chelator BAPTA-AM at concentration that effectively blocked S− induced TFEB nuclear localization significantly reduced TFEB nuclear translocation in PE-treated cells (Fig. 3e). This result suggests the importance of cytosolic Ca^2+^ for PE-induced TFEB activation.

Altogether, our findings demonstrate the activation of TFEB by PE is independent of the reported ERK1/2, mTORC1 and calcineurin. Instead, the mechanism underscoring PE-mediated TFEB activation represents a novel regulatory pathway that requires cytosolic Ca^2+^.

### PE induces “donut-shaped” mitochondria and mitophagosome formation basally to potentiate efficient mitophagy during mitochondrial stress

Mitochondrial stress has been reported to stimulate mitophagy via TFEB activation^26,29,30^. Similarly in our study, addition of mitochondrial uncoupler carbonyl cyanide m-chlorophenyl hydrazone (CCCP) reduced the levels of P-TFEB^S142^ and induced TFEB nuclear localization (Supplementary Fig. S4a). Interestingly, PE is ~4 times more potent than CCCP in activating TFEB (Supplementary Fig. S4a). This prompted us to examine the role of PE-induced autophagy in mitochondrial clearance.

**Figure 4:**
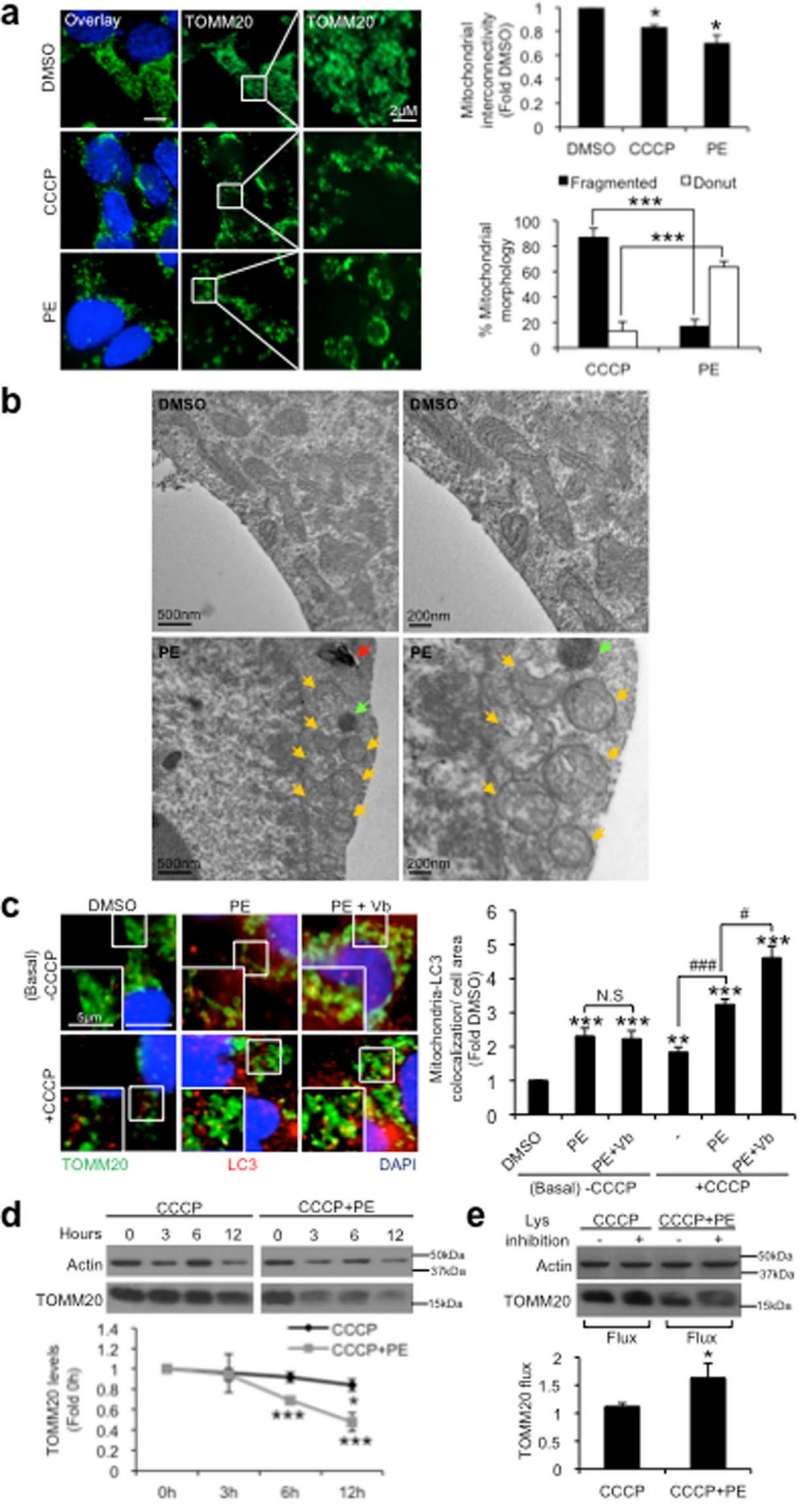
PE alters mitochondrial morphology basally and enhances mitophagosome formation to facilitate mitophagy under CCCP stress. (**a**) Left: Immunofluorescence images of TOMM20 in SY5Y cells treated with DMSO, 10 µM CCCP or 300 μg/ml PE for 16 h. Representative views of the different types of mitochondrial morphology are shown in the smaller inlets. Right: Quantification of mitochondrial interconnectivity (Top) and percentage of cells containing fragmented or “donut” mitochondrial morphology (Bottom). Mitochondrial interconnectivity is expressed as fold change relative to DMSO. (**b**) Electron micrographs depicting different mitochondrial morphology in SY5Y cells treated with DMSO or 300 μg/ml PE for 24 h. Donut- and C-shaped (partial donut) mitochondria are highlighted by yellow arrows. Autophagic vacuoles (red arrow) and lysosomes (green arrow) are found in close proximity to the donut/C-shaped mitochondria. (**c**) Left: Immunofluorescence images of TOMM20 and LC3 colocalization in SY5Y cells treated with DMSO, 300 µg/ml PE or PE with 1 µM vinblastine (Vb), in the absence (Basal/-CCCP) or presence of 10 µM CCCP for 16 h. Smaller inlets show close-up views of colocalization between TOMM20 and LC3. Right: Quantification of TOMM20 and LC3 colocalization per cell area, calculated as fold change against DMSO. Nuclei were stained with DAPI. 20 cells from random fields were analyzed for mitochondrial interconnectivity and TOMM20-LC3 colocalization. (**d**) Top: Immunoblot of TOMM20 in SY5Y cells treated with 10 µM cycloheximide (CHX), over a 12 h time-course treatment with 10 μM CCCP in the absence or presence of 300 μg/ml PE. Bottom: Quantification of TOMM20 levels at each time point, expressed as fold change against 0 h. Full-length blots are presented in Supplementary Figure S9c. (**e**) Top: Immunoblot of TOMM20 in SY5Y cells treated with 10 µM cycloheximide (CHX) and subjected to 10 μM CCCP alone or supplemented with 300 μg/ml PE in the absence or presence of lysosomal inhibition (NL with 1 µM Vb) for 12 h. Bottom: Quantification of CCCP-induced TOMM20 flux in the absence or presence of PE, expressed as TOMM20 levels under lysosomal inhibition against untreated condition. Full-length blots are presented in Supplementary Figure S9d. All values are mean + S.E.M (n = 3–7). Differences against DMSO or CCCP treatment only are significant at *p < 0.05, **p < 0.01, ***p < 0.005, ^#^p < 0.05 and ^###^p < 0.005. N.S = Not significant. Scale bar is 10 μm, unless otherwise stated.

Mitophagy involves alteration of mitochondrial morphology, by enhancing mitochondrial fission to isolate the damaged organelles from the remaining healthy network for autophagic removal^31^. We first examined whether PE influences the mitochondrial morphology basally as an indication of mitophagy. The mitochondria were stained with anti-TOMM20 antibody and the interconnectivity of the organelle was measured using the “Mito-morphology” Image J macro program^32^. In line with reported finding^33^, CCCP reduced the interconnectivity of the mitochondrial network by causing significant mitochondrial fragmentation (Fig. 4a). PE treatment similarly reduced the mitochondrial interconnectivity (Fig. 4a). However, unlike CCCP, PE led to an enrichment of characteristic “donut-shaped” mitochondria (Fig. 4a) with lowered mitochondrial ROS production (Supplementary Fig. S4b). Electron microscopy analysis revealed high percentage of spherical mitochondria, with some enclosing a cavity, and C-shaped mitochondria (intermediary structure to spherical conformation) in cells treated with 300 µg/ml PE for 24 h (Fig. 4b), corroborating the donut-shaped mitochondria observed by fluorescence microscopy. Autophagic vacuole and lysosome can be seen in the vicinity of the spherical/C-shaped mitochondria (Fig. 4b). In contrast, mitochondria in DMSO control cells are pleomorphic and mostly in tubular form. Understanding of the significance of “donut-shaped” mitochondria is currently limited. However, we were keen to investigate whether this morphological change influences the autophagic susceptibility of the mitochondria basally. By co-staining with anti-TOMM20 and anti-LC3 antibodies, the interaction between the mitochondria and the autophagosome was examined. Compared to DMSO control cells, ~1.3-fold higher LC3 colocalization with TOMM20 was observed in PE-treated cells basally (Fig. 4c). Biochemical analysis likewise indicated an enhanced LC3-II levels associated with purified mitochondria isolated from PE-treated cells under basal condition (Supplementary Fig. S4c). These results demonstrate that the “donut-shaped” mitochondria in PE-treated cells promote recruitment of autophagosomes (or formation of mitophagosomes) basally. Interestingly, addition of autophagy inhibitor vinblastine (Vb) to the PE-treated cells did not further enhance the colocalization between LC3 and TOMM20 basally (Fig. 4c). These results demonstrate that the enhanced formation of mitophagosomes observed with PE supplementation does not lead to mitochondrial degradation under basal condition.

We next seek to understand if PE may instead, potentiate mitophagy during mitochondrial stress. Enhanced LC3 and TOMM20 colocalization was observed under CCCP stress (Fig. 4c), consistent with the known notion of CCCP as a mitophagy inducer^34^. PE supplementation under CCCP stress further enhanced the association between LC3 and TOMM20 (Fig. 4c). Additionally, Vb caused an overt accumulation of mitophagosomes in PE-treated cells during CCCP stress (Fig. 4c). Biochemical analysis likewise indicated an accumulation of LC3-II in the mitochondrial fraction of PE-treated cells under CCCP-induced stress, with the levels further increased upon lysosomal inhibition with NL (Supplementary Fig. S4d). These results demonstrate that unlike basal condition, PE promotes formation of mitophagosomes to potentiate mitophagy during mitochondrial stress.

The enhanced mitophagy phenomenon in PE-treated cells under mitochondrial stress is further supported by the immunoblot analysis of TOMM20 levels overtime under CCCP stress. To streamline the effects on TOMM20 to enhanced mitophagy and not biogenesis changes, cells were co-treated with protein synthesis inhibitor cycloheximide (CHX) and CCCP (Supplementary Fig. S4e). CCCP treatment led to the progressive decline in TOMM20 levels over time, with significant reduction of ~10% seen at the 12 h timepoint (Fig. 4d). However, addition of PE dramatically accelerated the rate of TOMM20 clearance under CCCP stress, where ~30% TOMM20 reduction was detected at the 6 h timepoint (Fig. 4d). To affirm the faster TOMM20 clearance was caused by enhanced mitophagy stimulated by PE, we measured the TOMM20 flux upon autophagy inhibition with NL in the absence of protein synthesis. Comparatively, PE supplementation induced ~0.5 fold more accumulation of TOMM20 when compared to CCCP stress alone upon autophagy inhibition (Fig. 4e). Clearly, PE contributes to quality control of damaged mitochondria during stress by increasing the rate of mitochondrial autophagic removal.

### PE increases PINK1-Parkin mitophagy during mitochondrial stress

**Figure 5:**
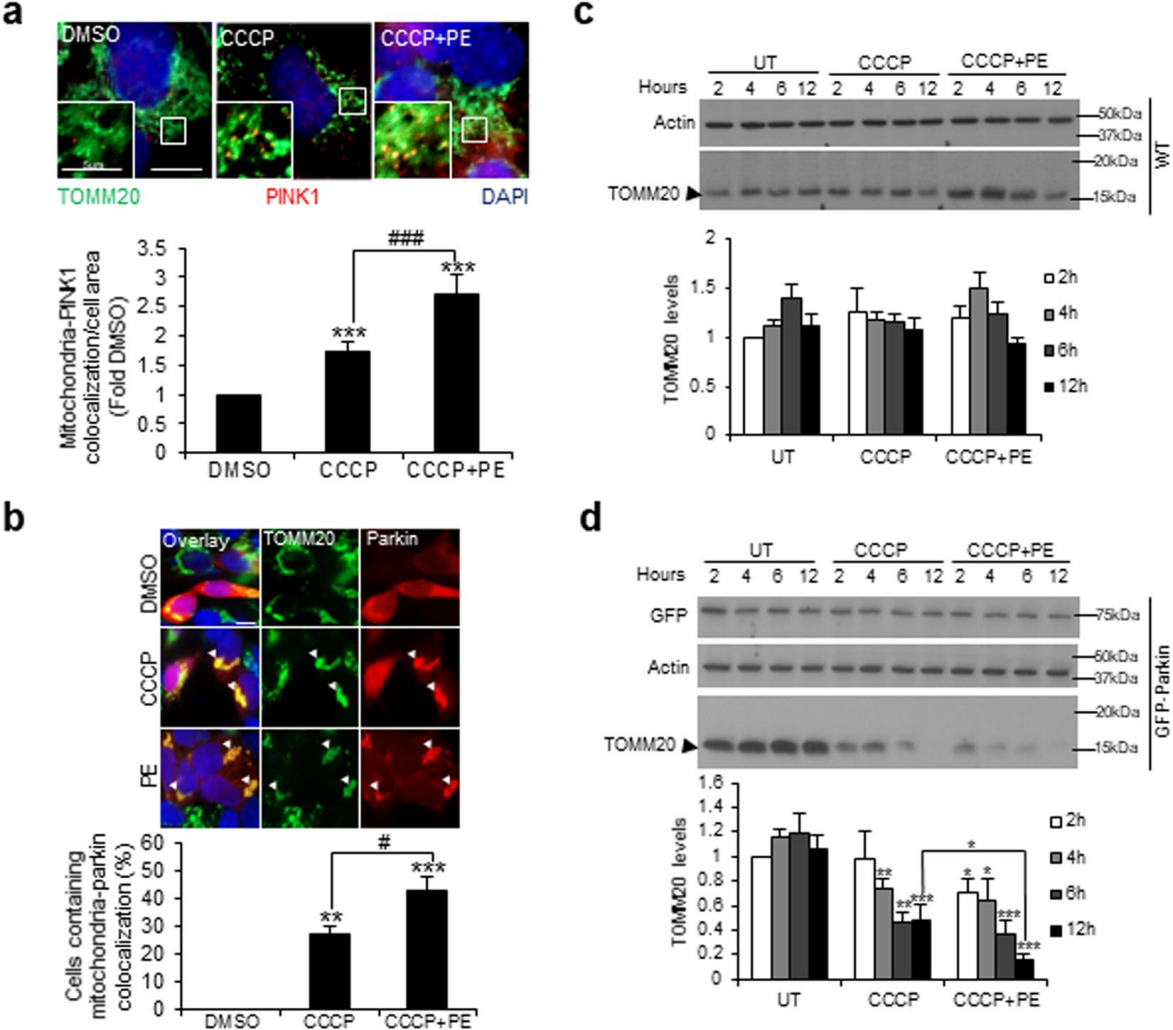
PE enhances the presence of PINK1 and Parkin on mitochondria to potentiate PINK1-Parkin dependent mitophagy under CCCP-induced stress. (**a**) Top: Immunofluorescence images of TOMM20 and PINK1 colocalization in SY5Y cells treated with DMSO, 10 µM CCCP or CCCP with 300 μg/ml PE for 16 h. Smaller inlets show close-up views of TOMM20 and PINK1 colocalization. Bottom: Quantification of TOMM20 and PINK1 colocalization per cell area, expressed as fold change against DMSO control. (**b**) Top: Immunofluorescence images of TOMM20 in mCherry-Parkin SY5Y stable cells treated with DMSO or 10 µM CCCP in the absence or presence of 300 μg/ml PE for 6 h. White arrows depict the colocalization between mitochondria aggregates and Parkin. Bottom: Quantification of percentage cells containing TOMM20 and Parkin colocalization. 20–30 cells from random fields were analyzed for the colocalization. Nuclei were stained with DAPI. (**c**,**d**) Top: Immunoblots of TOMM20 in WT (**c**) and GFP-Parkin (**d**) HeLa stable cells left untreated (UT), or treated with 10 µM CCCP or CCCP supplemented with 300 µg/ml PE for 2 h, 4 h, 6 h and 12 h. Bottom: Quantification of TOMM20 levels across the various treatment timepoints, expressed as fold change against 2 h UT condition. Full-length blots are presented in Supplementary Figure S10c and d. All values are mean + S.E.M (n = 3–7). Differences against DMSO, UT or CCCP are significant at *p < 0.05, **p < 0.01, ***p<0.005, ^#^p < 0.05 and ^###^p < 0.005. Scale bar is 10 μm, unless otherwise stated.

To elucidate the mechanism underlying PE-driven mitophagy during CCCP stress, we examined the involvement of PINK1 and Parkin, the two well-characterized mitophagy players that mediate mitochondrial clearance^34^. First, we examined how PE influences the recruitment of PINK1 and Parkin to the mitochondria, by monitoring the colocalization between PINK1/Parkin and TOMM20. Basally, PE supplementation has negligible effect on PINK1 and TOMM20 colocalization (Supplementary Fig. S5). This is consistent with the earlier observation that PE does not facilitate basal mitophagy. On the other hand, CCCP treatment significantly increased PINK1-TOMM20 colocalization by ~0.7-fold, and this was further enhanced upon co-treatment with PE (Fig. 5a). Similarly, addition of PE almost doubled the levels of colocalization between Parkin and TOMM20 than CCCP alone in mCherry-Parkin SY5Y stable cells (Fig. 5b). Evidently, PE enhances PINK1 and Parkin recruitment to the mitochondria during CCCP stress.

To validate the enhanced mitochondrial recruitment of mitophagy players during CCCP stress induced by PE was indeed associated with mitophagy, we employed GFP-Parkin HeLa stable cells to assess how PE influences mitochondrial clearance in the absence and presence of Parkin. In WT HeLa cells that do not express Parkin endogenously^35,36^, neither CCCP nor addition of PE affected the TOMM20 levels across the various treatment timepoints (Fig. 5c). In contrast, significant reduction in the TOMM20 levels was observed upon 4 h, 6 h and 12 h CCCP treatment in the GFP-Parkin HeLa stable cells (Fig. 5d). These observations are consistent with the notion that Parkin mediates mitophagy under CCCP stress. Under CCCP stress, further addition of PE led to an earlier reduction in the TOMM20 levels upon 2 h treatment when compared with CCCP alone in the stable cells (Fig. 5d). Notably, the reduction in TOMM20 levels upon 12 h CCCP treatment was further enhanced with PE (Fig. 5d). These results show that PE not only induces earlier, but also enhances the magnitude of mitochondrial turnover in the presence of Parkin under mitochondrial stress. These data show that PE enhances mitophagy via PINK1-Parkin pathway during mitochondrial stress.

### PE promotes turnover of dysfunctional mitochondria and attenuates mitochondrial redox toxicity

**Figure 6:**
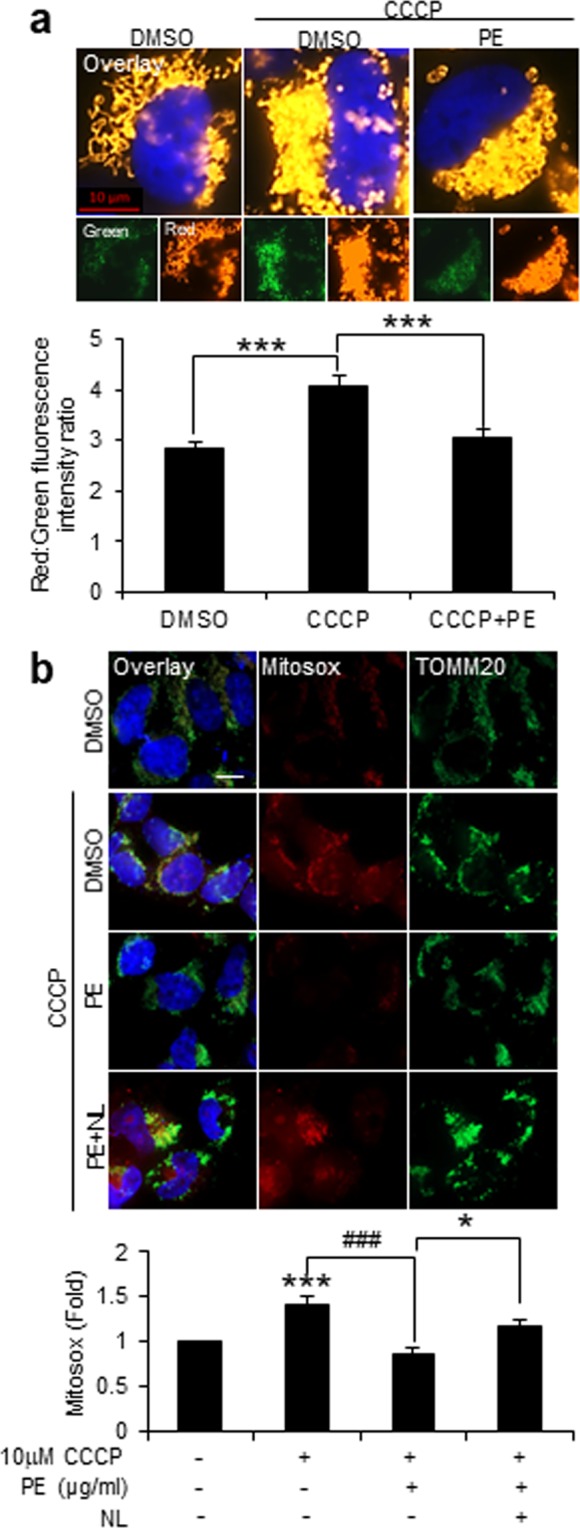
PE reduces damaged mitochondria and alleviates mitochondrial redox toxicity. (**a**) Top: Fluorescence images of SY5Y cells stably expressing MitoTimer reporter treated with DMSO or 10 μM CCCP in the absence or presence of 300 μg/ml PE for 16 h. Smaller inlets show the green (young mitochondria) and red (aged and damaged mitochondria) MitoTimer expression under each condition. Bottom: Quantification of the average red:green intensity ratio of MitoTimer per cell. (**b**) Top: Measurement of mitochondrial ROS via Mitosox staining and TOMM20 immunofluorescence in SY5Y cells treated with DMSO, 10 μM CCCP alone or 10 μM CCCP in the presence of 300 μg/ml PE or 300 μg/ml PE supplemented with lysosomal inhibitor (NL) for 16 h. Bottom: Quantification of Mitosox intensity level per cell area, expressed as fold change against DMSO. At least 30 cells from random fields were analyzed for MitoTimer and Mitosox assays. Nuclei were stained with DAPI. All values are mean + S.E.M (n = 3–7). Differences against DMSO, CCCP or CCCP + PE are significant at *p < 0.05, ***p < 0.005 and ^###^p < 0.005. Scale bar, 10 μm.

Dysfunctional mitochondria produce high levels of ROS that leads to intracellular redox toxicity^37^. Hence, we evaluated the effectiveness of PE-induced mitophagy to protect the cellular milieu from harmful mitochondrial ROS during mitochondrial stress. We first ascertained if PE selectively targets the damaged mitochondria for degradation by monitoring the response of MitoTimer. The MitoTimer is a mitochondria-targeted reporter for mitochondrial oxidative stress and damage, where its fluorescence shifts irreversibly from green to red following oxidation events^38^. CCCP treatment significantly increased the ratio of red:green MitoTimer fluorescence by ~1-fold, which indicates an increased levels of mitochondrial stress and damage (Fig. 6a). Remarkably, PE supplementation reduced and reverted the ratio induced by CCCP to basal DMSO control level (Fig. 6a). This demonstrates that PE promotes autophagic clearance of damaged mitochondria to possibly preserve mitochondrial health under stress condition.

Next, we investigated the protectiveness of PE-induced mitophagy against toxic ROS generated by damaged mitochondria using Mitosox staining. An overt increase in the Mitosox signals was observed with CCCP treatment, and this was significantly reduced in the presence of PE supplementation (Fig. 6b). However, inhibition of autophagy with NL abolished PE protection against Mitosox accumulation under CCCP stress (Fig. 6b). This highlights that PE-induced mitophagy attenuates superfluous mitochondrial ROS production by promoting clearance of damaged mitochondria.

### TFEB knockdown abolishes PE-induced autophagy and protection against mitochondrial dysfunction

**Figure 7:**
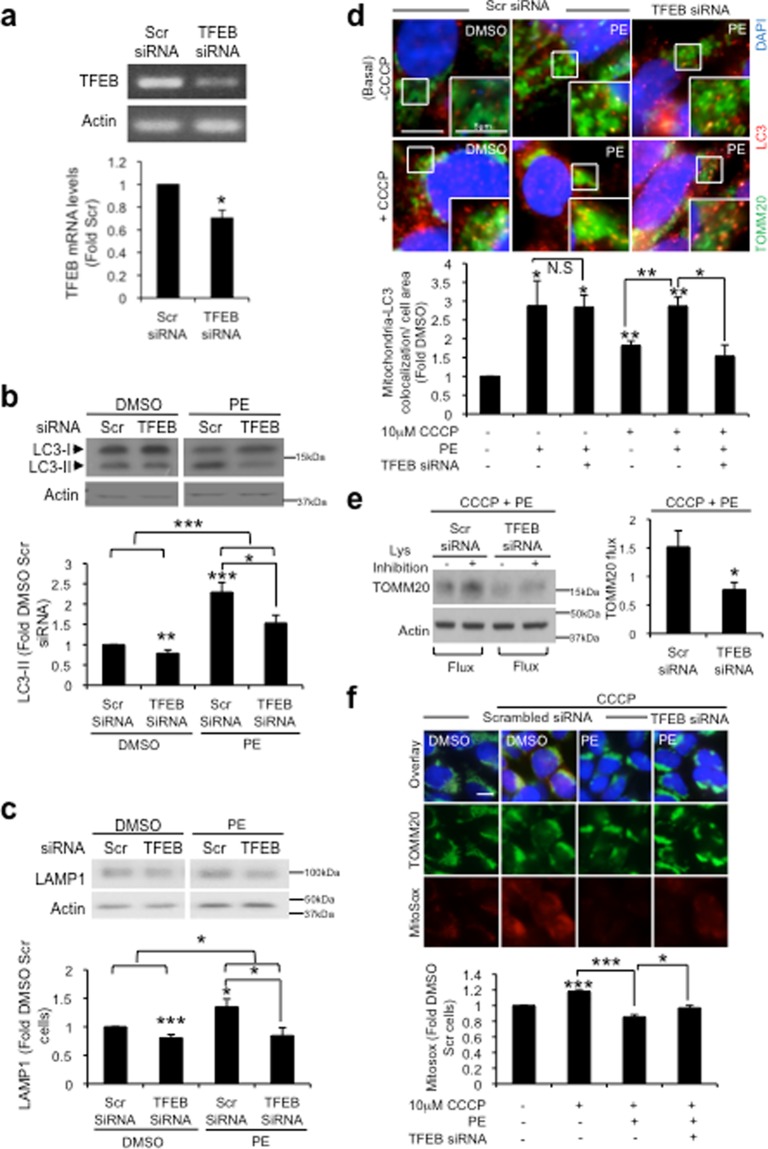
siRNA-mediated TFEB knockdown (KD) abolishes PE-induced autophagy and protection against mitochondrial ROS stress in SY5Y cells. (**a**) Top: PCR analysis of TFEB mRNA expression levels in SY5Y cells after 72 h transfection with 25 μM scrambled (Scr) siRNA or TFEB siRNA. Bottom: Quantification of TFEB mRNA levels, expressed as fold change against Scr control. (**b-c**) Top: Immunoblots of LC3 (**b**) and LAMP1 (**c**) in Scr control and TFEB KD cells treated with DMSO or 300 μg/ml PE for 16 h. Bottom: Quantification of LC3-II (**b**) and LAMP1 (**c**) levels, expressed as fold change against Scr siRNA control cells treated with DMSO. Full-length blots are presented in Supplementary Figure S11b and c. (**d**) Top: Immunofluorescence images of TOMM20 and LC3 colocalization in Scr siRNA control and TFEB KD cells, untreated or treated with 10 μM CCCP supplemented with either DMSO or 300 μg/ml PE for 16 h. Bottom: Quantification of TOMM20 and LC3 colocalization per cell area, expressed as fold change against DMSO treated Scr siRNA control cells. (**e**) Left: Immunoblot of TOMM20 in CHX-treated Scr siRNA control and TFEB KD cells subjected to 10 µM CCCP and 300 μg/ml PE in the absence or presence of lysosomal inhibition (NL + 1 µM Vb). Right: Quantification of TOMM20 flux in Scr siRNA control and TFEB KD cells. Full-length blots are presented in Supplementary Figure S11e. (**f**) Top: Immunofluorescence images of TOMM20 and Mitosox in Scr siRNA control cells treated with DMSO, 10 µM CCCP or CCCP with 300 µg/ml PE, and in TFEB KD cells treated with 10 µM CCCP with 300 µg/ml PE. Bottom: Quantification of Mitosox intensity per cell area, expressed as fold change against DMSO treated Scr siRNA control cells. 20–30 cells from random fields were analyzed for TOMM20-LC3 colocalization and Mitosox levels. Nuclei were stained with DAPI. All values are mean + S.E.M (n = 3–7). Differences are significant at *p < 0.05, **p < 0.01, ***p < 0.005. N.S = Not significant. Scale bar is 10 μm, unless otherwise stated.

Our results thus far indicate that PE upregulates mitophagy to negate mitochondrial stress. To verify that these homeostatic benefits were indeed dependent on TFEB, TFEB expression was abolished via siRNA-mediated knockdown (KD) (Fig. 7a) and the consequences were examined. We first assessed the effects of TFEB KD on PE ability to enhance the autophagy-lysosomal compartments. In DMSO treated cells, TFEB KD significantly reduced the levels of both LC3-II (Fig. 7b) and LAMP1 (Fig. 7c) when compared to scrambled (Scr) control. This is consistent with the fact that TFEB is the master transcriptional regulator of the autophagy-lysosomal pathway^13^. In PE-treated cells, the robust upregulation of LC3-II (Fig. 7b) and LAMP1 (Fig. 7c) levels were attenuated with TFEB KD. Densitometric analysis revealed that the reduction in LC3-II and LAMP1 caused by TFEB KD were significantly greater in PE-treated cells than DMSO control cells (Fig. 7b and c). This result shows that loss of TFEB function has a more pronounced effect in PE-treated cells, which could be attributed to the upregulation of autophagy-lysosomal compartments by PE. Thus, TFEB activation is integral to PE ability to augment the autophagy-lysosomal compartments.

Next, we examined the consequences of TFEB KD on PE-induced mitophagy and protection against mitochondrial stress. We first looked at the formation of mitophagosomes. Basally, TFEB KD has no effect on the enhanced LC3 and TOMM20 colocalization mediated by PE (Fig. 7d). However, KD of TFEB abrogated the enhanced TOMM20-LC3 colocalization induced by PE under CCCP stress (Fig. 7d). These results demonstrate that unlike under basal condition, the recruitment of autophagosomes to mitochondria induced by PE under mitochondrial stress is dependent on TFEB. In addition, loss of TFEB function significantly reduced TOMM20 flux during CCCP stress by ~50% as compared to Scr control cells (Fig. 7e). These findings support that PE activates TFEB to augment mitophagosomes formation and mitophagy under stress condition.

Lastly, we investigated if the loss of mitophagy caused by TFEB ablation reduces PE protection against mitochondrial ROS during CCCP insult. While clear reduction in Mitosox signal was observed with PE supplementation under CCCP stress, the reduction was partially prevented in TFEB KD cells (Fig. 7f). These findings show that activation of TFEB by PE accords protection against overt mitochondrial ROS generation and malfunction during stress condition. Taken together, PE activates TFEB to upregulate autophagic compartments for efficient mitophagy during mitochondrial stress and negates mitochondrial damage.

## Discussion

Autophagy dysregulation is a feature commonly observed in aging and age-related disorders like neurodegeneration^39,40^. The critical role of TFEB as a master regulator of genes involved in the lysosomal–autophagic pathways makes it an attractive therapeutic target for human diseases associated with autophagy or lysosomal dysfunction. Indeed, an increasing number of studies have demonstrated the potential of TFEB induction in mitigating lysosomal storage disorders^41–44^ and proteinopathies^45–49^. In addition, TFEB activation is associated with healthy lifespan and longevity^50,51^. Therefore, molecules or compounds that can activate TFEB hold promise as interventions to delay the onset or progression of aging and diseases.

Currently known TFEB activators involve stress triggers such as starvation and mitochondrial stress, as well as mTOR inhibitors^52^. However, mTOR regulates numerous cellular processes and inhibition of the kinase is likely to elicit a plethora of undesirable side effects^53^. Thus, methods to activate TFEB without affecting the mTOR signaling are preferred. Recently, a synthetic analog of the polyphenol curcumin has been shown to activate TFEB without influencing mTOR^16^. In this regard, we have identified PE as another natural alternative to potentiate autophagy via TFEB activation independent of mTOR signaling. Pomegranate and its constituents have been shown to induce autophagy in different cells lines, including glioblastoma, syncytiotrophoblast, papillary thyroid and hepatocellular carcinoma cells^21–23,54^. Our finding re-emphasizes the universal nature of PE to induce autophagy in various cell types. PE supplementation increases TFEB nuclear localization (Fig. 2a and b), accompanied by the enhancement of transcriptional program for the autophagy-lysosomal pathway (Fig. 2c) and autophagy activity (Fig. 1). In fact, PE exhibits surpassing effectiveness in activating TFEB compared to starvation and CCCP stressors (Fig. 2a and Supplementary Fig. S4a). Owing to its profound effect on TFEB activation, PE supplementation poses as a viable strategy to prevent or delay age-related pathologies by improving the cellular autophagy-lysosomal fitness. This idea is supported by the observation that PE supplementation in *C. elegans* and drosophila improves healthspan and lifespan by ensuring better growth, oxidative stress and infection resistance^10,11^.

Interestingly, PE-induced TFEB activation is independent of the known TFEB modulators, namely ERK1/2^25^, mTORC1^17–19,25^ and calcineurin^20^ (Fig. 3). Rather, TFEB activation by PE appears to depend on intracellular Ca^2+^ levels (Fig. 3e). Ca^2+^ is a ubiquitous second messenger with wide-ranging physiological roles^55^. Many of the Ca^2+^-mediated events occur when the ion binds to and activate the effector protein. In our study, the Ca^2+^ effector calcineurin appears to be dispensable for PE-induced TFEB activation (Fig. 3d), suggesting that Ca^2+^ modulates TFEB activity via other regulatory mechanism that remains to be determined. PE may influence yet identified calcium-binding proteins to either downregulate TFEB phosphorylation or upregulate TFEB dephosphorylation to activate TFEB. Nonetheless, our study unravels a novel mechanism for TFEB activation that is independent of the known ERK1/2, mTORC1 and calcineurin pathways.

TFEB participates in a myriad of cellular processes^13^. In this study, PE upregulates TFEB-induced autophagy for mitochondrial quality control. Under basal condition, PE distinctively influences basal tubular mitochondrial network and encourages formation of “donut-shaped” mitochondria (Fig. 4a,b). Understanding of the significance of “donut-shaped” mitochondria is currently limited. Studies have proposed that the “donut” shape is an intermediate configuration between tubular shape and mitochondrial fragmentation^56^. Spherical mitochondria are observed to form rapidly and transiently during the early response to mitochondrial depolarization in a fission-independent way^57,58^. The “donut” mitochondria either derived from mitochondrial membrane bending around cytoplasmic constituents or from the invagination of mitochondrial membrane to form a cavity to pull in cytoplasm^57,58^. Studies have shown that organelles such as vesicles and endoplasmic reticulum (ER) can be enclosed by or pulled into the cavity of the “donut” mitochondria. This could explain the enhanced association between the “donut” mitochondria and autophagosomes upon PE treatment under basal condition. Ring-shaped mitochondria are found to exist in normal tissues but they are more frequently seen in stress conditions^58^. The “donut” mitochondria can revert back to its healthy, tubular configuration upon removal of stress, or proceed irreversibly to fragmentation to elicit mitophagic response under prolonged stress^56,59^.

Analysis of the intrinsic properties of the “donut” mitochondria revealed that these structures have lowered respiratory capacity and higher mitochondrial ROS generation^56^. This reported finding markedly contrasted with our observation, where the “donut” mitochondria observed basally with PE supplementation have lowered ROS level and therefore, reflects a healthier mitochondrial state (Supplementary Fig. S4b). Thus, we think that the “donut” mitochondria induced by mitochondrial stress and PE have different properties and functions. The “donut’ configuration formed during mitochondrial stress may serve as a protective conformation to temporally prevent the mitochondria from progressing towards terminal mitochondrial fragmentation and irreversible toxicity. On the contrary, PE driven “donut” configuration is not due to stress. In fact, the antioxidant property of PE aids in reducing mitochondrial ROS levels. We postulate that PE-mediated intracellular Ca^2+^ changes may bring about mitochondrial membrane potential changes or curvature to form spherical mitochondria. Further, PE enhances the recruitment of the autophagosomes to the “donut” mitochondria basally during the structural transformation to form the spherical mitochondria (Fig. 4c). This enhanced formation of mitophagosomes basally is not associated with mitochondrial turnover but resembles a primed state on standby to facilitate efficient mitophagy upon onset of stress (Fig. 4c). This is further supported by the enhanced recruitment of autophagosomes to the mitochondria induced by PE during CCCP-induced stress (Fig. 4c). Subsequently, this cellular occurrence stimulates higher mitochondrial turnover than CCCP-induced mitophagy alone (Fig. 4d,e). These results demonstrate that PE induces optimal autophagosome positioning to coordinate efficient mitophagy upon onset of mitochondrial stress. The coordinated positioning of autophagosomes and lysosomes to prime for efficient autophagic degradation has also been reported in other cellular stress conditions such as starvation and proteotoxic stress^60^. For example, in response to proteasomal dysfunction, lysosomes redistribute from the cell periphery to the perinuclear region to facilitate efficient autophagic removal of protein aggregates^61^. It will be interesting to explore whether PE also orchestrates the redistribution of autophagic organelles under other cellular stress conditions to prime autophagy induction.

Interestingly, PE localizes PINK1 and Parkin to the mitochondria under CCCP stress (Fig. 5a,b). The presence of Parkin further augments CCCP-induced mitophagy with PE supplementation, as overexpression of Parkin in Parkin-deficient HeLa cells enhanced mitochondrial protein TOMM20 clearance in the presence of PE under CCCP stress (Fig. 5c,d). These findings demonstrate that PE mediates stress-induced mitophagy via the PINK1-Parkin pathway. Altogether, our findings show that PE orchestrates TFEB activation to potentiate PINK1-Parkin stress-induced mitophagy to mitigate mitochondrial dysfunction. Of note, it will be interesting to explore whether Parkin KD in SY5Y cells attenuates PE-induced mitophagy under CCCP stress.

Several reports demonstrate TFEB as a downstream effector of Parkin for mitophagy induction^29,30,62^. However, the mechanism underlying PE-induced mitophagy appears to work in the opposite manner, where PE centrally activates TFEB to initiate downstream mitophagy event during mitochondrial stress. This is supported by the observations where TFEB KD abolishes mitophagosome formation (Fig. 7d), mitochondrial clearance (Fig. 7e) and protection against superfluous mitochondrial ROS generation (Fig. 7f).

Mitophagy is a key determinant of longevity where it protects against age-dependent accruement of deleterious mitochondria to reduce futile ATP hydrolysis, ROS production, pro-inflammatory responses and mutated mitochondrial DNA burden^63–66^. Defects in mitophagy have also been observed in pathologies such as cancer, metabolic syndrome and neurodegeneration^3,4,64,67^. Hence, PE-induced mitophagy poses as a viable way to improve mitochondrial health and to prolong lifespan and prevent age-related pathologies. In support of this, urolithin A, a metabolite of PE-associated polyphenol ellagitannins, induces mitophagy and extends healthspan and lifespan in *C. elegans*^12^. Urolithin A-induced mitophagy was also recapitulated in mammalian muscle and intestinal cells, which is shown to improve muscle functions in rodents by preventing age-related accumulation of dysfunctional mitochondria and decline in metabolic bioenergetics^12^. Similar to PE, urolithin A also alters mitochondrial morphology^12^. Although the mechanism that underpins urolithin A-induced mitophagy remains obscure, our study strongly suggests TFEB as a mediator of urolithin A and PE-induced mitophagy.

**Figure 8:**
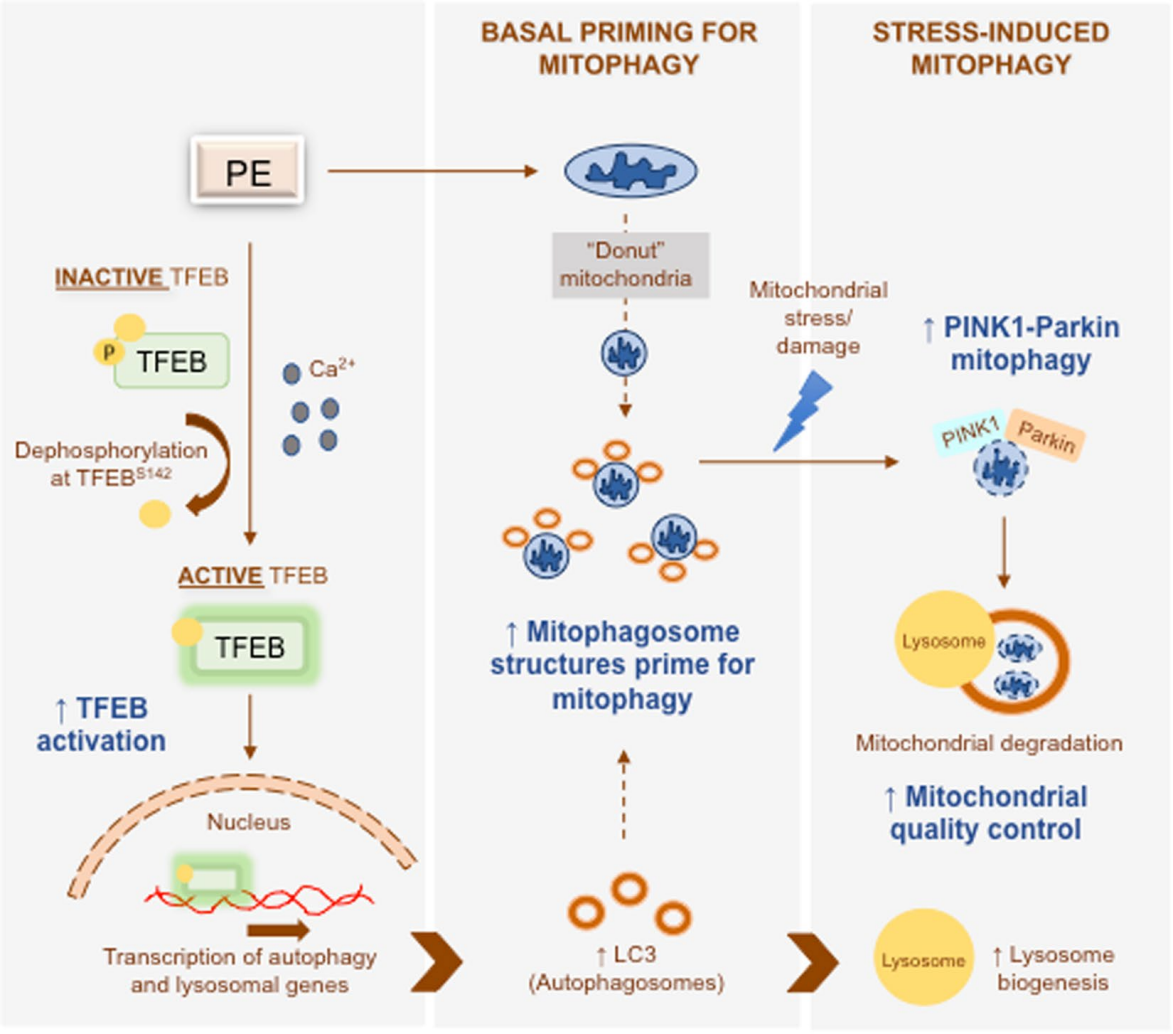
PE activates TFEB to prime mitochondria for efficient mitophagosome formation basally and potentiate PINK1-Parkin mitophagy during mitochondrial stress. PE reduces phosphorylation of TFEB at S142 via a mechanism that depends on cytosolic Ca^2+^. Dephosphorylated TFEB (active TFEB) rapidly translocates to the nucleus, where it mediates transcription of autophagy and lysosomal genes to increase the cellular pools of autophagosomes and lysosomes. The enhanced levels of autophagosomes associate with the mitochondria basally, aided by the “donut-shaped” mitochondrial configuration induced by PE. PE does not promote mitochondrial degradation basally but instead makes mitochondria highly competent to undergo efficient mitophagy when needed. PE primes mitochondria for timely mitophagosome formation in face of severe mitochondrial stress to rapidly remove damaged mitochondria by increasing the recruitment of autophagosomes to mitochondria. Upon onset of mitochondrial stress, PE facilitates the recruitment of PINK1 and Parkin to the mitochondria to initiate PINK1-Parkin dependent mitophagy. This occurrence promotes efficient mitochondrial degradation and quality control during mitochondrial stress to preserve mitochondrial health.

In summary, our study demonstrates PE as an activator of TFEB (Fig. 8). PE centrally activates TFEB to globally enhance the transcriptional program of the autophagy-lysosomal pathway, via a mechanism that requires cytosolic Ca^2+^ (Fig. 8). Upregulation of autophagy by PE is associated with mitochondrial quality control. Basally, PE promotes recruitment of autophagosomes to “donut-shaped” mitochondria (Fig. 8). This phenomenon primes the mitochondria on standby for efficient mitophagy through timely mitophagosome formation (Fig. 8). Under mitochondrial stress, PE potentiates PINK1-Parkin mitophagy to counteract the accumulation of dysfunctional mitochondria and superfluous ROS production in a TFEB dependent manner (Fig. 8). Taken together, activation of TFEB by PE positively influences mitochondrial health. Hence, PE supplementation represents a potential therapeutic strategy for mitochondrial-related diseases.

## Methods

### Constructs and antibodies

GFP-TFEB, MitoTimer, mCherry-Parkin and blasticidin-resistance expression plasmids were from Addgene plasmid repository. Antibodies and their respective dilutions used in immunoblotting (IB) and immunofluorescence (IF) were: AKT (C67E7) (Cell Signaling Technology (CST), IB: 1:1000); β-actin (Abcam, IB: 1:10,000); β-tubulin (Developmental Studies Hybridoma Bank (DSHB), IB: 1:2000); ERK (clone 16) (BD Bioscience, IB: 1:1000); GFP (Roche, IB: 1:3000); LAMP1 (H4A3) (DSHB, IB: 1:1000, IF: 1:300); LC3 (CST for IB, 1:1000; Novus for IF, 1:300); mTOR (CST, IB: 1:1000); p70/S6K (CST, IB: 1:1000); PINK1 (Abcam, IF: 1:500); phospho-AKT^T308^ (CST, IB: 1:1000); phospho-AKT^S473^ (CST, IB: 1:1000); phospho-mTOR^S2448^ (CST, IB: 1:1000); phospho-p44/42 MAPK (ERK1/2)^T202/Y204^ (CST, IB: 1:1000); phospho-p70/S6K^T389^ (CST, IB: 1:1000); phospho-TFEB^S142^ (Millipore, IB: 1:3000); TOMM20 (Abcam, IB: 1:1000, IF: 1:1000). For IB, mouse and rabbit horseradish peroxidase (HRP) conjugated secondary antibodies (Sigma) were used at 1:10,000 dilution. For IF, Alexa Fluor-conjugated secondary antibodies (Invitrogen) were used at 1:800 dilution.

### Inhibitors

The following chemicals and drugs were used to inhibit the autophagy pathway: 20 mM ammonium chloride (NH_4_Cl) (Sigma) and 100 μM leupeptin (Enzo) to inhibit the lysosome activity; 1 μM (IF) or 10 μM (IB) vinblastine (Sigma) to inhibit the fusion between autophagosome and lysosome. 10 µM FK506 (Sigma) was used to inhibit the phosphatase calcineurin and 10 μM BAPTA-AM (Invitrogen) was used to chelate the cytosolic Ca^2+^. To inhibit protein translation and synthesis, 10 μg/ml cycloheximide (CHX) (Sigma) was used.

### PE

PE was prepared from GNC Herbal Plus® Standardized Pomegranate powdered capsule (GNC, Singapore) dissolved in 5 ml dimethyl sulfoxide (DMSO) (Sigma) to yield 5 mg/ml PE stock. Working concentrations for PE were reconstituted by diluting the stocks in culture media. DMSO was used as the solvent vehicle control.

### Cell culture and transfection

Human neuroblastoma SY5Y cells were cultured in Dulbecco’s Modified Eagle’s Medium (DMEM) (Invitrogen) supplemented with 10% FBS (GE Healthcare) and 1% penicillin-streptomycin (Thermo Fisher Scientific). All the cell lines were grown in 5% CO_2_ at 37 °C. For transfection, cells were transfected using Lipofectamine® 2000 (Invitrogen) at a 1:3 DNA to lipofectamine ratio following manufacturer’s protocol.

### Generation of mCherry-Parkin, GFP-TFEB and MitoTimer SY5Y stable cells

To generate the various stable cell lines, SY5Y cells were co-transfected with 1.0 μg plasmid of interest and 1.0 μg blasticidin-resistance vector for 72 h. Thereafter, co-transfected cells expressing both plasmids were selected by 10 μg/ml blasticidin (Invitrogen) treatment for 5–7 days. Positive clones stably expressing the respective proteins were subsequently cultured in 2 μg/ml blasticidin.

### siRNA knockdown of TFEB and RT-PCR analysis

SY5Y cells were transfected with 25 μM scrambled siRNA or TFEB siRNA for 72 h. Human TFEB siRNA target sequence is 5′-CCGCCTGGAGATGACCAACAA-3′ (Qiagen Hs_TFEB-2). Efficiency of siRNA-mediated silencing of TFEB was checked by RT-PCR analysis. RNA was extracted using Ambion Purelink RNA mini kit (Life Technologies) followed by DNase treatment with RQ1 RNase-Free DNase (Promega) according to manufacturer’s protocol. Isolated RNA was subsequently reverse transcribed using M-MLV Reverse Transcriptase (Promega) and amplified with GoTaq Flexi DNA Polymerase (Promega) for agarose gel analysis. The following RT-PCR primers were used for TFEB: Forward primer 5′ GTAGGACTGCACCTTCAACACCT-3′; Reverse primer 5′-TCACGCATAGGGTTGCGCAT-3′. To investigate calcineurin gene expression, the RNA was isolated and reverse transcribed same as above. The following RT-PCR primers were used for calcineurin: Forward primer 5′-GCTGCCCTGATGAACCAAC-3′; Reverse primer 5′-GCAGGTGGTTCTTTGAATCGG-3′. β-actin was used as a housekeeping control for TFEB and calcineurin gene expression. The following RT-PCR primers were used for β-actin: Forward primer 5′-CCAGAGGCGTACAGGGATAG-3′; Reverse primer 5′-CCAACCGCGAGAAGATGA-3′.

### Autophagy PCR array

The Human Autophagy RT^2^ Profiler PCR Array (Qiagen) was used to study autophagy-specific gene expression profiles in accordance with the manufacturer’s recommendations. Briefly, RNA was isolated, DNase treated and reverse transcribed as discussed in “siRNA knockdown of TFEB and RT-PCR analysis”. Real-time PCR was performed with the RT² SYBR Green qPCR Mastermix (Qiagen) and Applied Biosystems 7500 standard qPCR machine according to the manufacturer’s instructions. The amplification data (fold change in Ct value of all the genes) were analyzed by the ΔΔCt method.

### Cell lysis, SDS-PAGE, immunoblotting and densitometry

*C*ells were lysed in radioimmunoprecipitation assay (RIPA) buffer (50 mM Tris-base pH 7.4, 1% Nonidet P-40, 0.5% sodium deoxycholate, 150 mM sodium chloride, 1 mM ethylenediaminetetraacetic acid (EDTA) supplemented with protease and phosphatase inhibitors (Roche)). After 15 min of lysis on ice, the cells were centrifuged at 14,000 rpm for 15 min at 4 °C to collect the supernatants for sodium dodecyl sulfate polyacrylamide gel electrophoresis (SDS-PAGE) and immunoblotting. Protein concentrations were determined with Bio-Rad Bradford Protein Assay (Bio-Rad) using bovine serum albumin (BSA) as a standard. Protein bands recognized by specific antibodies were visualized using Pierce ECL Western Blotting Substrate detection kit (Thermo Fisher Scientific). Densitometric analysis was performed using Image J software (NIH).

### Mitochondrial fractionation

Mitochondria were isolated from SY5Y cells as previously described^68,69^. Briefly, cells were washed with cold 1x PBS and lysed in homogenization medium (75 mM sucrose, 30 mM Tris-HCl, 0.1 mM EDTA, 225 mM mannitol, pH 7.4) followed by 5 min centrifugation at 1000 g. The supernatant was further centrifuged at 8000 g for 10 min. Upon removal of supernatant, the resultant mitochondrial pellet was resuspended in mitochondrial isolation medium (75 mM sucrose, 5 mM Tris-HCl, 225 mM mannitol, pH 7.4). Total lysed cells prior to separation were analyzed as total homogenate.

### Immunofluorescence

Cells grown on coverslips were fixed with 4% paraformaldehyde (PFA) and permeabilized with 0.1% Triton X-100 (Bio-Rad) prior to staining with the respective antibodies. Prolonged gold antifade medium containing DAPI (4′, 6-diamidino-2-phenylindole) (Invitrogen) was used during mounting. Images were acquired with Zeiss Z1 AxioObserver inverted fluorescence microscope (Zeiss) using 63x oil immersion objective lens. Random fields per slide were imaged and the same acquisition parameters were applied to the analysis of different treatment slides for fair comparison.

### Quantification of LC3, LAMP1 and mRFP-GFP-LC3 puncta

The number of LC3 and LAMP1 puncta were analyzed using the “analyze particles” function in Image J^70^. Briefly, the cell shape was outlined, and consistent threshold values were applied to highlight the puncta within the cell. Using the “analyze particles” function, the parameters for the puncta size were set at 0.1-infinity pixel^2^ and circularity at 0–1 for quantification. The puncta numbers were subsequently normalized against cell area. For tandem mRFP-GFP-LC3, the number of yellow puncta (autophagosomes) and red puncta (autophagolysosome) per transfected cell were quantified by manual counting and normalized against respective cell area measured using Image J program. For quantification of the cell area with Image J, the cell boundary was highlighted, and “measure” function was used to determine the area.

### Mitochondrial Interconnectivity

The interconnectivity of mitochondria was analyzed using an Image J “Mito-Morphology” macro (publicly available for download from the Image J Wiki site)^32^. Briefly, the cell shape was outlined, and consistent threshold value was applied to highlight the mitochondria within the cell. Using the “analyze particles” function, the highlighted mitochondrial structure will be measured for the mean area/perimeter ratio as an index of mitochondrial interconnectivity.

### Colocalization analysis

For colocalization analysis of LC3 and PINK1 with the mitochondria, cells were co-stained with anti-LC3 or anti-PINK1 with anti-TOMM20 antibody. A colocalization will be considered with the appearance of yellow punctate due to overlap between the LC3 or PINK1 (red) with the TOMM20 (yellow) signals. To confirm if the colocalization is real, each yellow punctate identified was checked against the position of the red and green signals in their respective channels to confirm the overlapped pattern. The number of colocalizations per cell was normalized against the cell area. The cell area was determined using the Image J program (refer to “Quantification of LC3, LAMP1 and mRFP-GFP-LC3 puncta”). 20 cells were analyzed per treatment condition.

For colocalization analysis of Parkin with the mitochondria, mCherry-Parkin SY5Y stable cells were stained with anti-TOMM20 antibody. A colocalization was scored if the mCherry signal (red) colocalized with the TOMM20 signal (green) with same morphology. 20–30 mCherry-Parkin stable cells were analyzed per treatment condition and the percentage of cells displaying colocalization was quantified.

### MitoTimer and Mitosox analysis

For analysis of mitochondrial health in MitoTimer stable cells, the fluorescence intensity of green (newly synthesized healthy mitochondria) and red (aged damaged mitochondria) MitoTimer proteins within each cell was quantified using the ZEN software (Zeiss). The mitochondrial health was expressed as the red: green intensity ratio. Mitochondrial ROS production was measured using Mitosox Red Mitochondrial Superoxide Indicator (Invitrogen) following manufacturer’s protocol. Briefly, cells were washed with pre-warmed 1x Hanks’ Balanced Salt Solution (HBSS) followed by staining with 3.75 μM Mitosox for 10 min at 37 °C. Cells were then washed with 1x HBSS and then co-stained with anti-TOMM20 antibody to highlight the mitochondrial morphology. Mitosox intensity per cell was analyzed using the ZEN software. For all analysis, 20–40 cells from random fields were analyzed.

### Electron microscopy

SY5Y cells treated with DMSO, 150 or 300 μg/ml PE for 24 h were fixed in 2.5% glutaraldehyde solution, embedded and sectioned for transmission microscopy imaging at the Electron Microscopy Unit facility at National University of Singapore, Singapore.

### Statistical analysis

For all quantitative analysis, results are shown as mean + standard error mean (S.E.M). Student’s t-test was used to determine statistical significance, defined by p value < 0.05.

## Supplementary information

Supplementary Figures

## Data Availability

All data generated or analyzed during this study are included in this published article (and its Supplementary Information files).
