# Supplementary material for: Pomegranate activates TFEB to promote autophagy-lysosomal fitness and mitophagy
Source: Sci Rep. 2019 Jan 24;9:727. doi: 10.1038/s41598-018-37400-1 (PMC6346015; doi:10.1038/s41598-018-37400-1)
Supplement: Supplementary file 1 — Supplementary Figures [file 41598_2018_37400_MOESM1_ESM.pdf]

Supplementary Information

Pomegranate activates TFEB to promote autophagy-lysosomal fitness and mitophagy

Sijie Tan[1], Chye Yun Yu[1], Zhi Wei Sim[1], Zun Siong Low[1], Brianna Lee[1], Faith See[1], Nyo Min[2], Archana Gautam[3], Justin Jang Hann Chu[2], Kee Woei Ng[3] and Esther Wong[1]*

Content:

This file contains the Supplementary Figures S1-11 and the accompanying figure legends.

Supplementary Figure S1

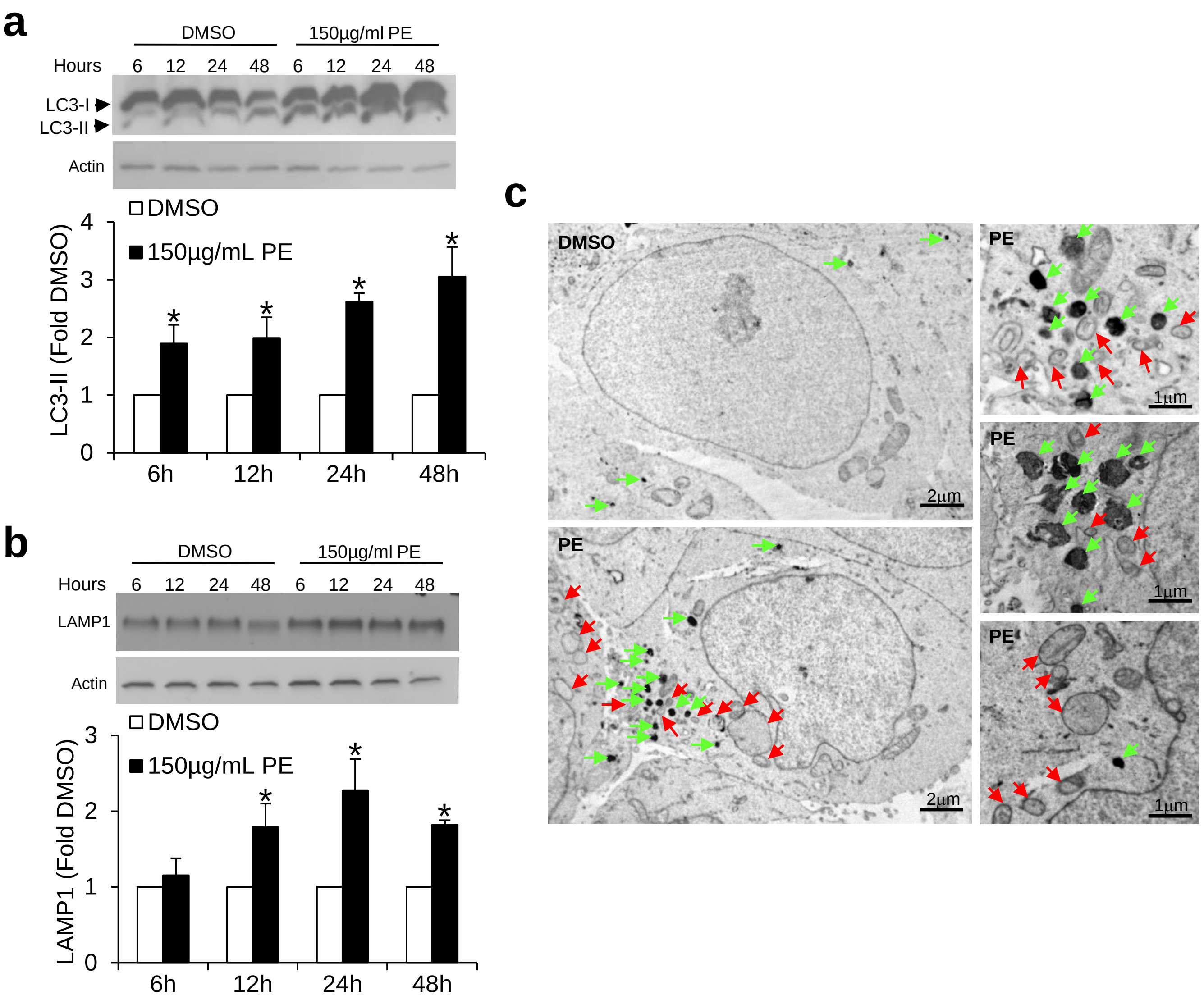

Supplementary Figure S1: PE upregulates autophagosomal and lysosomal compartments. (a-b) Top: Immunoblots of LC3 (a) and LAMP1 (b) in SY5Y cells treated with vehicle control DMSO and 150µg/ml PE for 6h, 12h, 24h and 48h. Bottom: Quantification of LC3-II and LAMP1 levels, calculated as fold change against DMSO control. All values are mean + S.E.M (n=3). Differences against DMSO control are significant at *$p<0.05$. (c) Electron micrographs of SY5Y cells treated with DMSO or 150µg/ml PE for 24h under basal condition. Higher magnification fields show autophagic vacuoles (red arrows) and electron dense lysosomes (green arrows).

Supplementary Figure S2

Gene Symbol	Fold Increase
AKT1	-0.49885
AMBRA1	2.034248 *
APP	-0.19742
ATG10	0.441079
ATG12	3.196264 *
ATG16L1	2.615564
ATG16L2	0.781141
ATG3	1.928958 *
ATG4A	1.886893 *
ATG4B	1.92071 *
ATG4C	-0.62582 *
ATG4D	4.080788
ATG5	2.193737
ATG7	1.673394
ATG9A	2.460342
ATG9B	3.271276
BAD	3.094935
BAK1	4.402007 *
BAX	1.651264
BCL2	2.865425*
BCL2L1	2.581412
BECN1	2.169702 *
BID	2.170097
BNIP3	6.982535 *
CASP3	2.159032
CASP8	2.5551
CDKN1B	1.583403
CDKN2A	1.800029 *
CLN3	1.49709 *
CTSB	2.489029
CTSD	1.34532 *
CTSS	0.585348
CXCR4	0.084326
DAPK1	1.613234 *
DRAM1	2.20426 *
DRAM2	1.769037 *
EIF2AK3	6.46106
EIF4G1	0.425993
ESR1	2.20931 *
FADD	1.400649 *
FAS	2.571388
GAA	-1.03225
GABARAP	1.671774
GABARAPL1	2.332196
GABARAPL2	1.60599 *
HDAC1	1.176462 *
HDAC6	0.487185
HGS	2.144028 *
HSP90AA1	0.639545

Supplementary Figure S2 (Continued)

Gene Symbol	Fold Increase
HSPA8	0.429459
HTT	2.051518
IFNG	2.831056
IGF1	1.23986
INS	1.37751
IRGM	0.934947
LAMP1	1.546296 *
MAP1LC3A	2.064182
MAP1LC3B	3.946444 *
MAPK14	1.421552
MAPK8	2.847527 *
MTOR	0.799862
NFKB1	2.880899
NPC1	1.935098 *
PIK3C3	2.166117 *
PIK3CG	1.143554 (n=2)
PIK3R4	1.498895
PRKAA1	2.404048 *
PTEN	1.306894
RAB24	2.394636 *
RB1	-0.99779
RGS19	1.128973 *
RPS6KB1	2.475376 *
SNCA	2.195009 *
SQSTM1	4.75271 *
TGFB1	1.496329
TGM2	0.108419
TMEM74	0.830013
TNF	5.010585
TNFSF10	0.295698
TP53	2.500337
ULK1	2.098623
ULK2	4.396573 *
UVRAG	3.006331 *
WIPI1	5.581288 *

Supplementary Figure S2: Real-time PCR profiling of 84 human autophagic genes upon PE treatment. Gene expression of 84 human autophagic genes in SY5Y cells treated with DMSO or 300µg/ml PE for 24h. The fold increase shown in the table is the average of at least 3 independent experiments. Genes whose expression are significantly down-regulated or up-regulated are indicated with the asterisk symbol * ($p < 0.05$).

Supplementary Figure S3

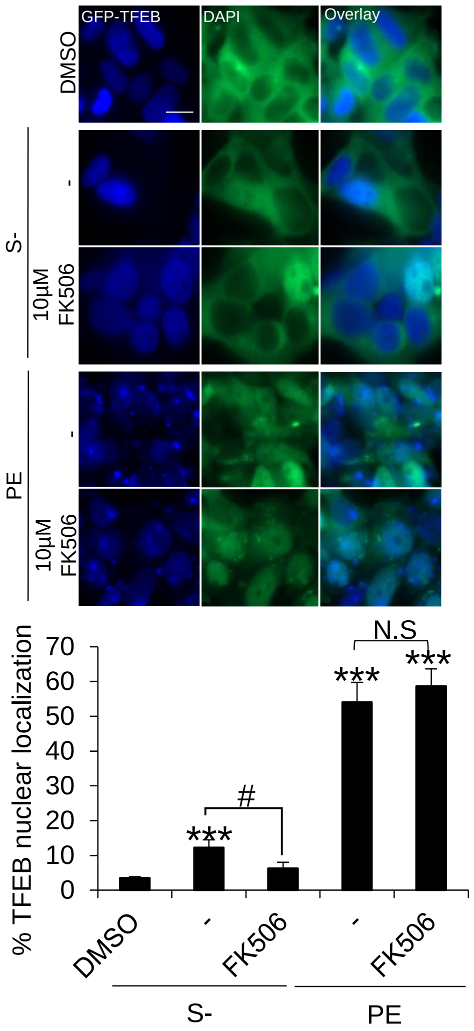

Supplementary Figure S3: PE modulates TFEB nuclear localization independent of calcineurin. Top: Fluorescence images depicting spatial localization of GFP-TFEB in GFP-TFEB SY5Y stable cells treated with DMSO, starved (S-), or treated with 300µg/ml PE in the absence or presence of 10µM calcineurin inhibitor FK506 for 16h. Nuclei were stained with DAPI. At least 100 cells from random fields were analyzed for each condition. All values are mean + S.E.M (n=3). Differences against DMSO, S- or PE are significant at ***p<0.005 and # p<0.05. N.S=Non-significant. Scale bar, 10µm.

Supplementary Figure S4

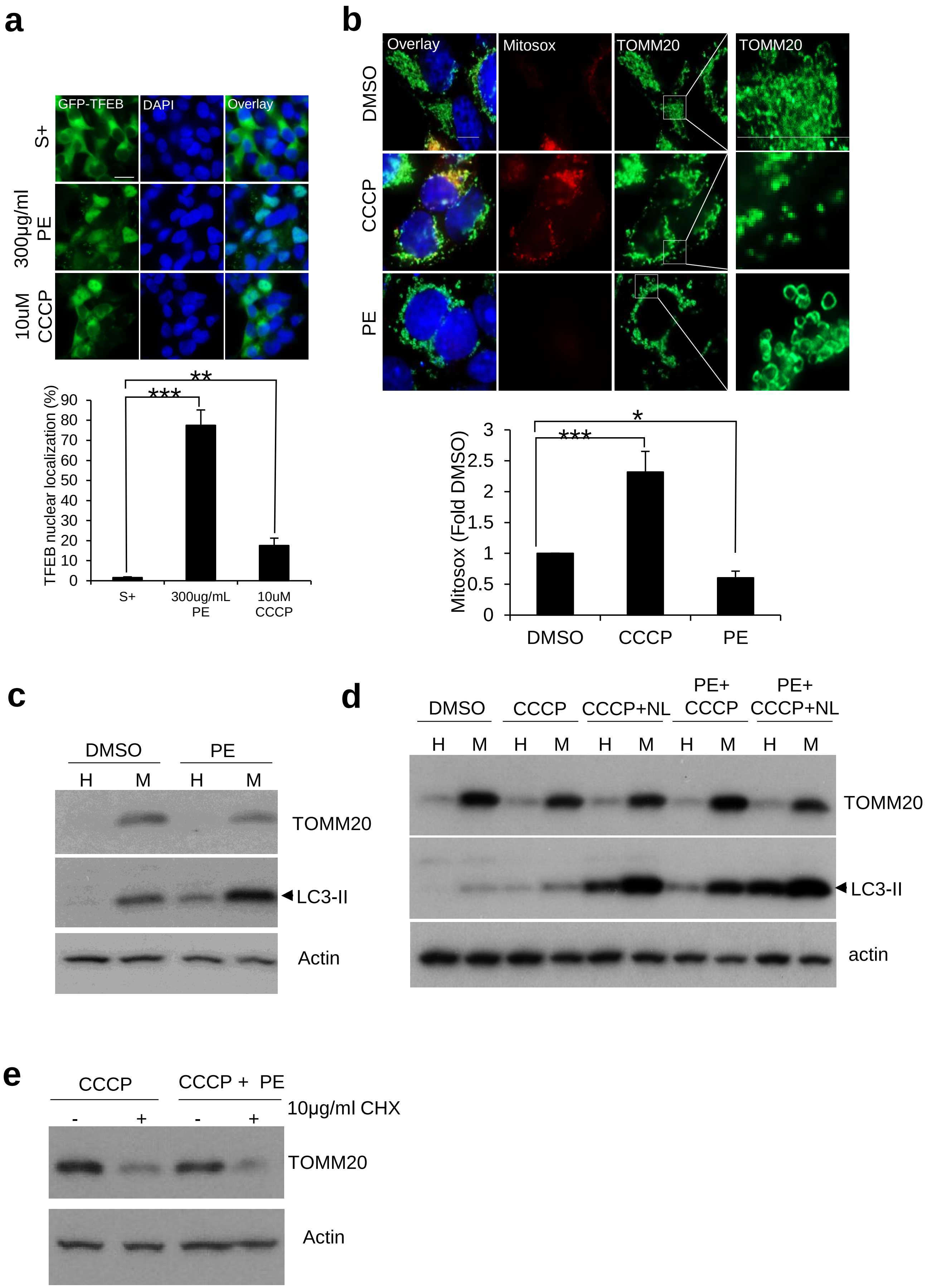

Supplementary Figure S4: (a) CCCP induces TFEB nuclear localization. Left: Fluorescence images depicting spatial localization of GFP-TFEB in GFP-TFEB SY5Y stable cells treated with DMSO, 300µg/ml PE or 10µM CCCP for 24h. Bottom: Quantification of percentage of cells containing nuclear-localized GFP-TFEB. **(b) PE-induced "donut" mitochondria has lowered mitochondrial ROS levels.** Top: Immunofluorescence images of Mitosox and TOMM20 staining in SY5Y cells treated with DMSO, 10µM CCCP and 300µg/ml PE for 16h. Smaller inlets show the close-up view of mitochondrial morphology under the different treatment conditions. Bottom: Quantification of Mitosox intensity level per cell area, expressed as fold change against DMSO. At least 100 cells from random fields were analyzed for TFEB nuclear localization, and at least 30 cells for Mitosox assay. Nuclei were stained with DAPI. Scale bar is 10µm unless otherwise stated. **(c-d) PE treatment enhances the presence of LC3-II in isolated mitochondria both basally and upon CCCP-induced stress.** (c) Immunoblots of TOMM20 and LC3 in total cell homogenate (H) and isolated mitochondrial fraction (M) prepared from SY5Y cells treated with DMSO or 300µg/ml PE 16h. (d) Immunoblots of TOMM20 and LC3 in total cell homogenate (H) and isolated mitochondrial fraction (M) in SY5Y cells treated with DMSO, 10µM CCCP alone or with lysosomal inhibitor (NL), and 10µM CCCP supplemented with 300µg/ml PE with or with lysosomal inhibitor (NL) for 16h. **(e) Cycloheximide treatment reduces mitochondrial TOMM20 protein synthesis.** Immunoblot of TOMM20 protein levels in SY5Y cells untreated or treated with 10µg/ml cycloheximide in the presence of 10µM CCCP alone or 10µM CCCP supplemented with 300 µg/ml PE for 12h. . All values are mean + S.E.M (n=3). Differences against S+ or DMSO PE are significant at *p<0.05, **p<0.01 and *** p<0.005.

Supplementary Figure S5

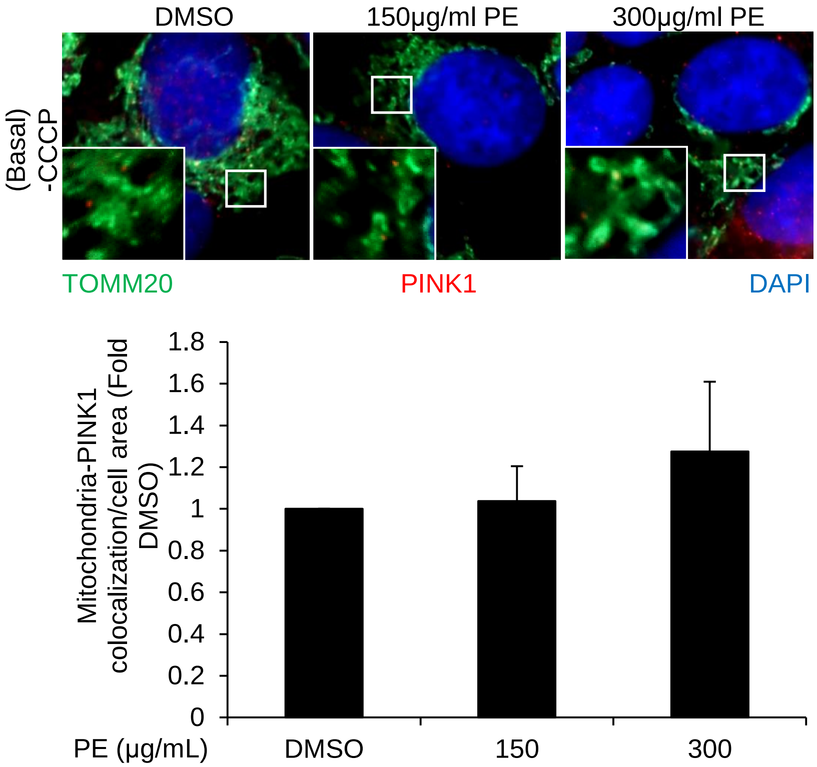

Supplementary Figure S5: PE does not induce PINK1 recruitment to the mitochondria under basal condition. Top: Immunofluorescence images of TOMM20 and PINK1 colocalization in SY5Y cells treated with DMSO, 150 or 300µg/ml PE for 16h. Smaller inlets show close-up views of TOMM20 and PINK1 colocalization. Bottom: Quantification of TOMM20 and PINK1 colocalization per cell area, expressed as fold change against DMSO. 20 cells from random fields were analyzed for the colocalization. Nuclei were stained with DAPI. All values are mean + S.E.M (n=3).

Supplementary Figure S6

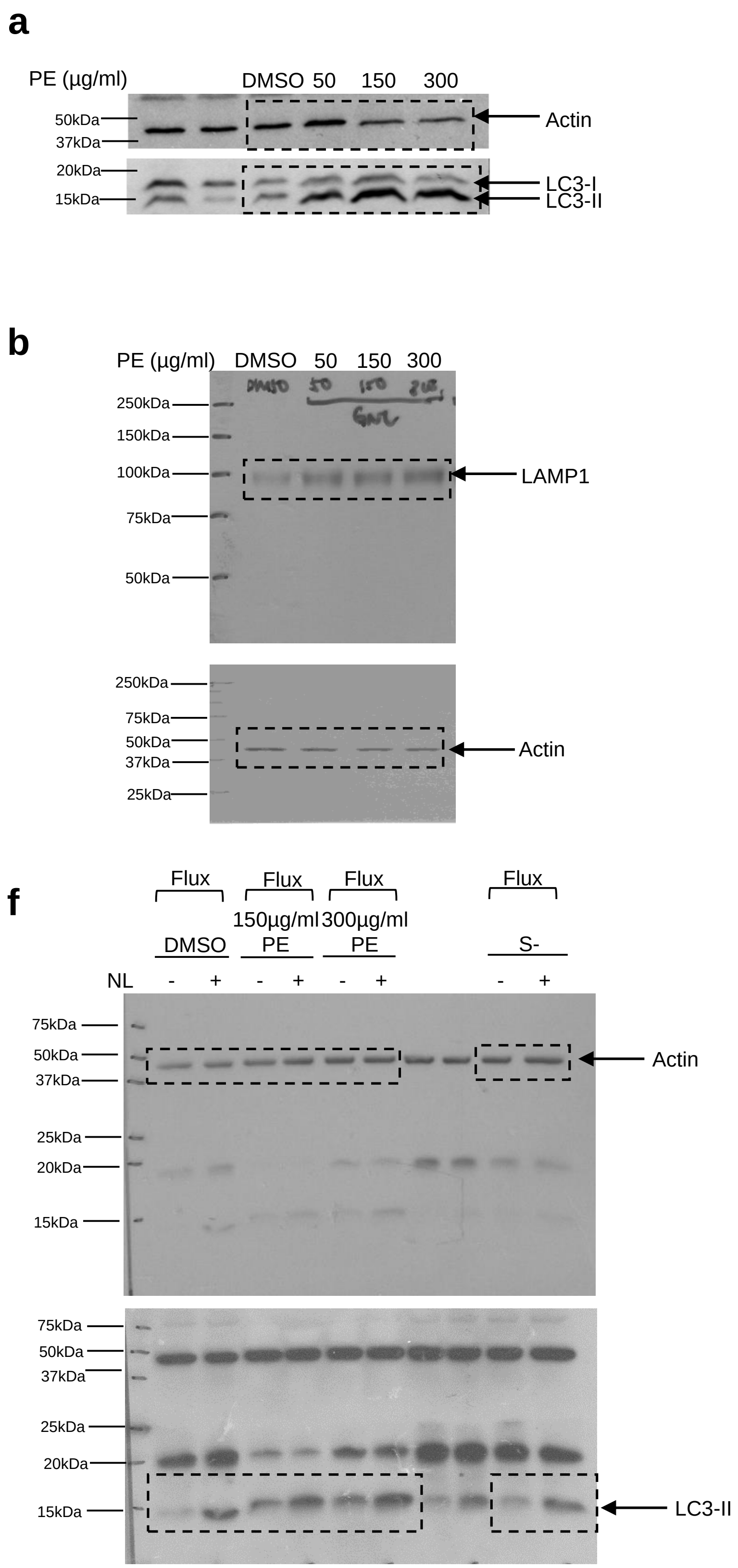

Supplementary Figure S6: Full-length blots corresponding to Fig. 1a, 1b and 1f.

Supplementary Figure S7

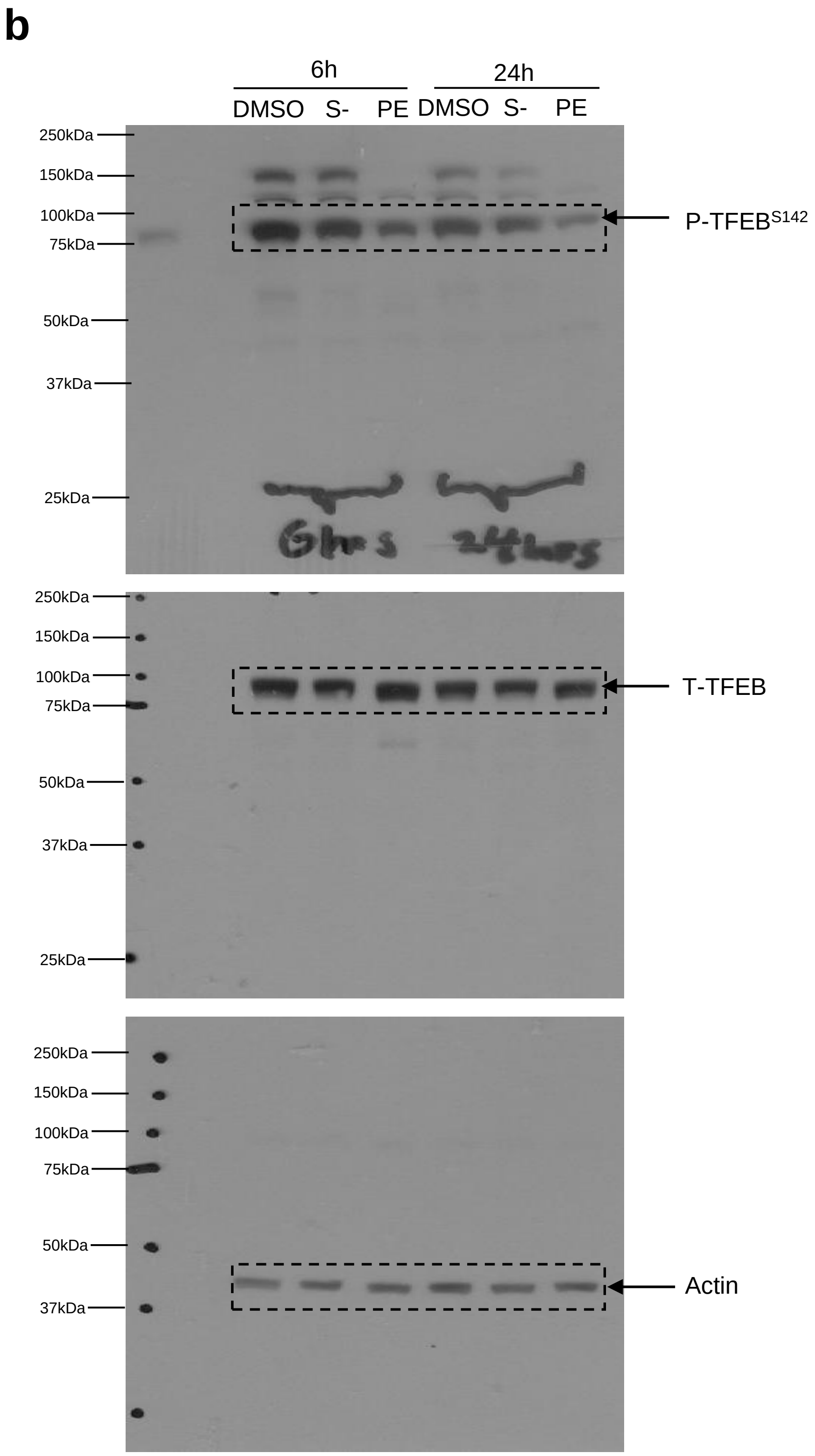

Supplementary Figure S7: Full-length blots corresponding to Fig.2b.

Supplementary Figure S8

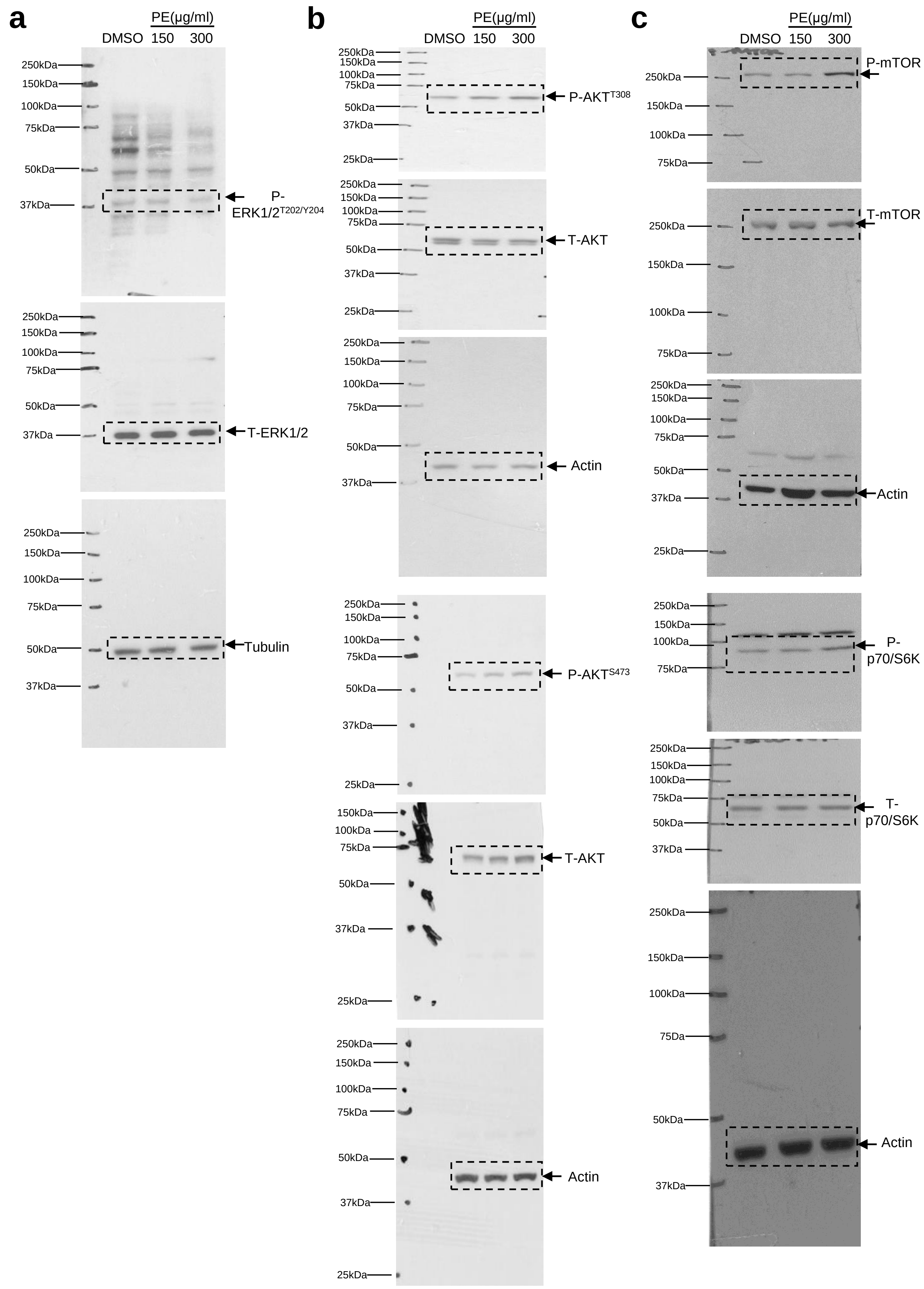

Supplementary Figure S8 (continued)

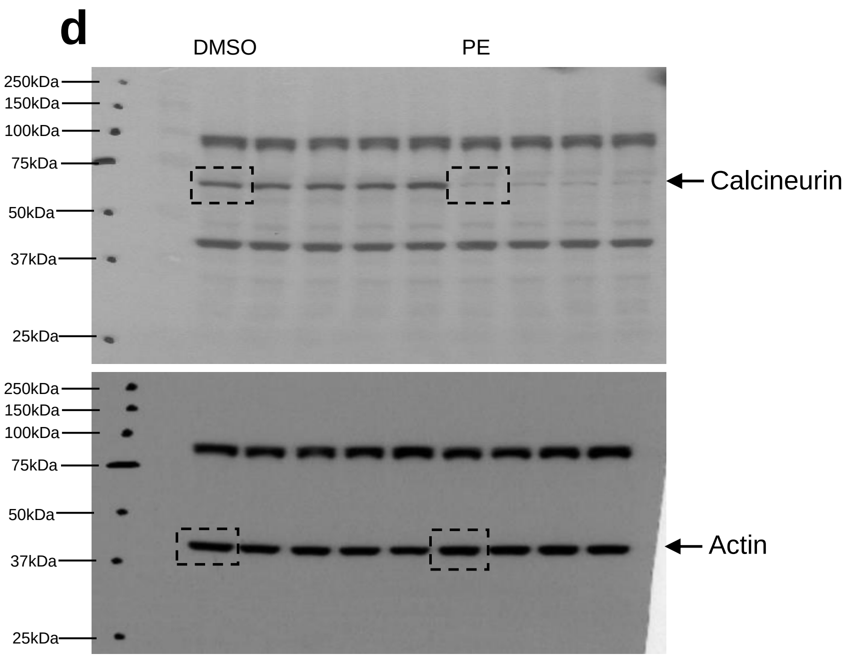

Supplementary Figure S8: Full-length blots corresponding to Fig. 3a-d.

Supplementary Figure S9

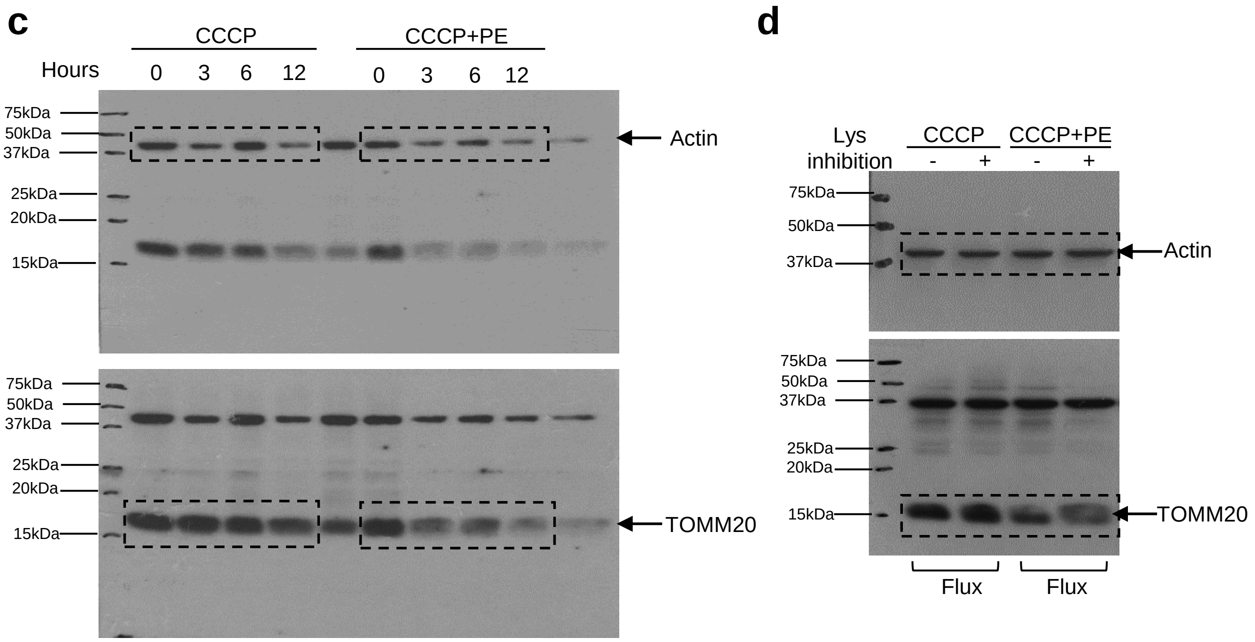

Supplementary Figure S9: Full-length blots corresponding to Fig. 4c and d.

Supplementary Figure S10

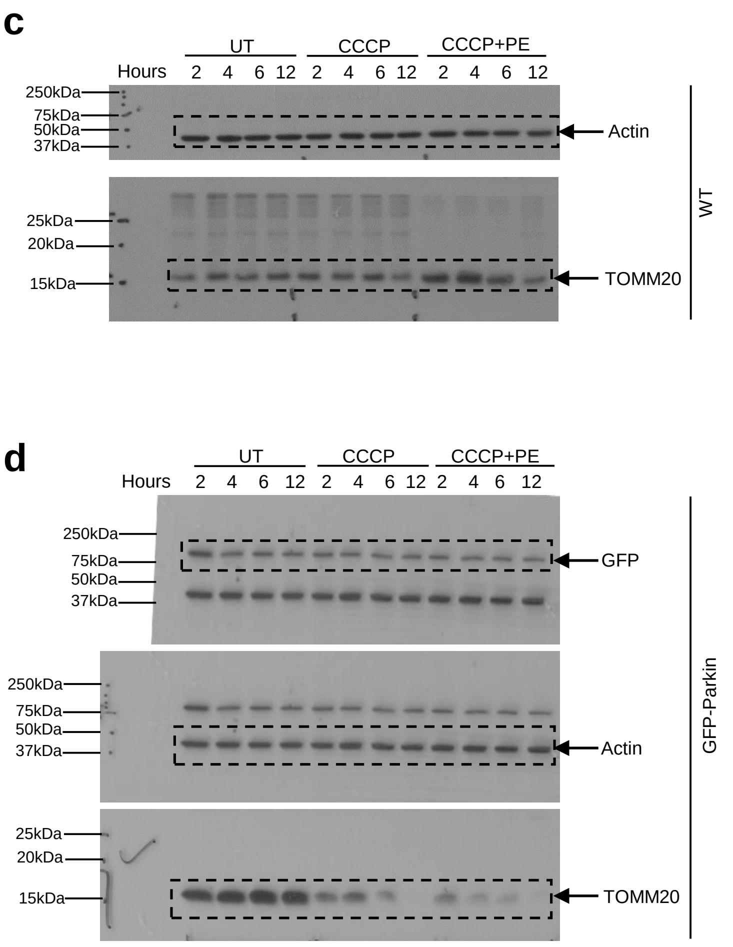

Supplementary Figure S10: Full-length blots corresponding to Fig. 5c and d.

Supplementary Figure S11

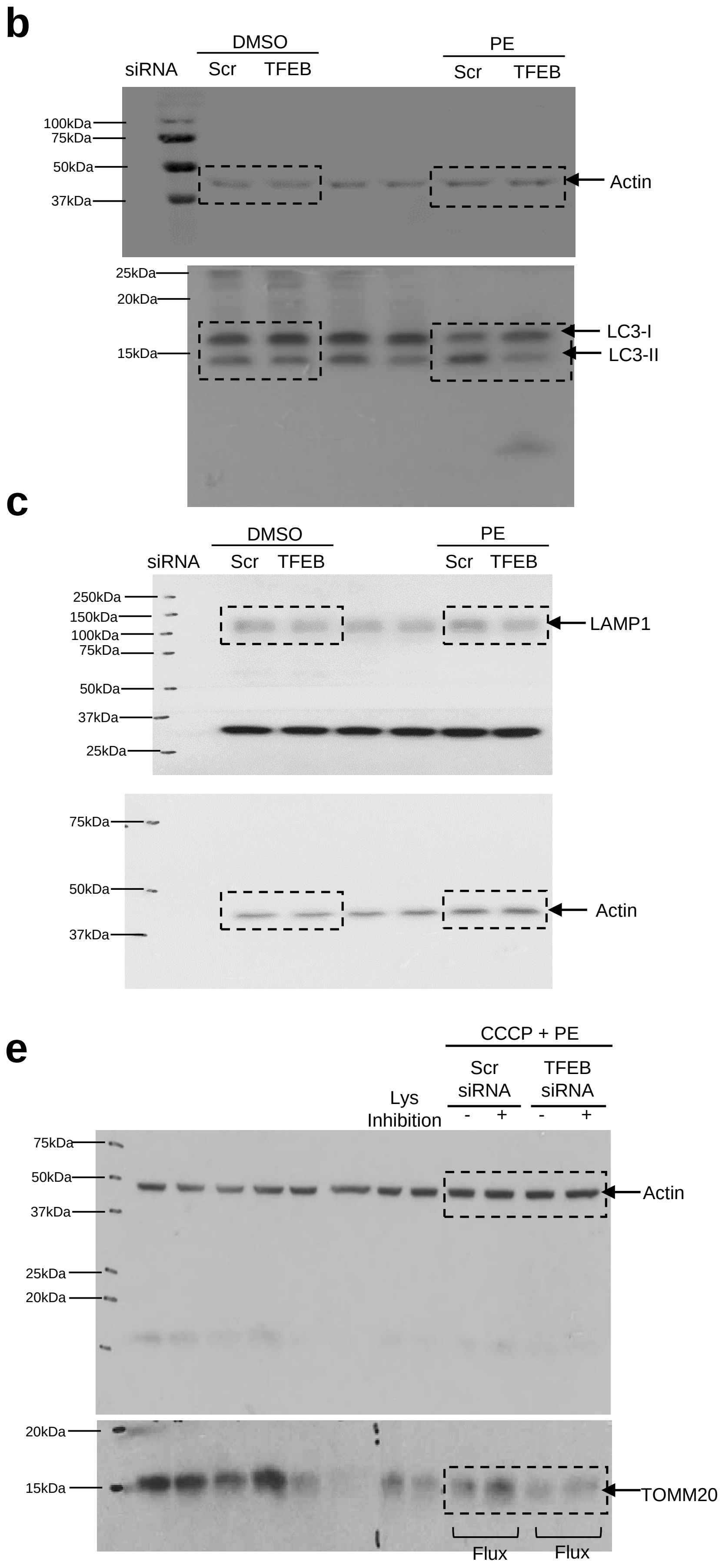

Supplementary Figure S11: Full-length blots corresponding to Fig. 7b-c and 7e.
